# Suppression of DS1 Phosphatidic Acid Phosphatase Confirms Resistance to *Ralstonia solanacearum* in *Nicotiana* *benthamiana*

**DOI:** 10.1371/journal.pone.0075124

**Published:** 2013-09-20

**Authors:** Masahito Nakano, Masahiro Nishihara, Hirofumi Yoshioka, Hirotaka Takahashi, Tatsuya Sawasaki, Kouhei Ohnishi, Yasufumi Hikichi, Akinori Kiba

**Affiliations:** 1 Laboratory of Plant Pathology and Biotechnology, Faculty of Agriculture, Kochi University, Nankoku, Kochi, Japan; 2 Iwate Biotechnology Research Center, Kitakami, Iwate, Japan; 3 Laboratory of Defense in Plant-Pathogen Interactions, Graduate School of Bioagricultural Sciences, Nagoya University, Chikusa-ku, Nagoya, Japan; 4 Division of Proteomedical Sciences, Cell-Free Science and Technology Research Center, Ehime University, Matsuyama, Japan; 5 Research Institute of Molecular Genetics, Kochi University, Nankoku, Kochi, Japan; University of Wisconsin-Milwaukee, United States of America

## Abstract

*Nicotiana* *benthamiana* is susceptible to *Ralstonia solanacearum*. To analyze molecular mechanisms for disease susceptibility, we screened a gene-silenced plant showing resistance to *R. solanacearum*, designated as DS1 (Disease suppression 1). The deduced amino acid sequence of *DS1* cDNA encoded a phosphatidic acid phosphatase (PAP) 2. *DS1* expression was induced by infection with a virulent strain of *R. solanacearum* in an *hrp*-gene-dependent manner*. DS1* rescued growth defects of the temperature-sensitive *∆lpp1∆dpp1∆pah1* mutant yeast. Recombinant DS1 protein showed Mg^2+^-independent PAP activity. DS1 plants showed reduced PAP activity and increased phosphatidic acid (PA) content. After inoculation with *R. solanacearum*, DS1 plants showed accelerated cell death, over-accumulation of reactive oxygen species (ROS), and hyper-induction of *PR-4* expression. In contrast, *DS1*-overexpressing tobacco plants showed reduced PA content, greater susceptibility to *R. solanacearum*, and reduced ROS production and *PR-4* expression. The DS1 phenotype was partially compromised in the plants in which both *DS1* and *NbCoi1* or *DS1* and *NbrbohB* were silenced. These results show that DS1 PAP may affect plant immune responses related to ROS and JA cascades via regulation of PA levels. Suppression of DS1 function or *DS1* expression could rapidly activate plant defenses to achieve effective resistance against *Ralstonia solanacearum*.

## Introduction

Plants have evolved sophisticated defense mechanisms that are activated in response to pathogen attacks. In most cases, plants resist infection through active defense mechanisms or the lack of compatibility with a given pathogen. The front line of induced defense is triggered by pathogen-associated molecular patterns (PAMPs), also known as PAMP-triggered immunity. PAMPs are generally conserved compounds, like chitin in fungi and flagellins in bacteria, and PAMP-triggered immunity is induced by all invading pathogens [1,2,3]. The second line of plant defense is activated via recognition of pathogen effectors by Resistance gene products, followed by triggering of effector-triggered immunity. Knowledge about disease resistance has increased tremendously in recent years. Numerous resistance genes have been cloned and many defense-associated and signal transduction genes have been identified [4,5]. However, little is known about the molecular basis of disease susceptibility.

A well-characterized example of a plant disease susceptibility factor is the transmembrane MLO protein. This protein has been identified as a negative regulator in *PEN* gene-associated disease resistance to powdery mildews [6,7,8]. In barley and *Arabidopsis* , loss-of-function mutations in *MLO* result in efficient pre-invasion resistance to adapted powdery mildews [9,10,11]. *Arabidopsis* loci required for susceptibility to *Erysiphecichoracearum* include *PMR6*, which encodes a pectate lyase-like protein [12,13], *PMR4*, which encodes a callose synthase [14], and *PMR5*, encoding a protein of unknown function [15]. The downy mildew-resistant (dmr) mutants *dmr1, dmr2*, and *dmr6* showed resistance in the absence of enhanced defense responses, suggesting that the corresponding genes are required for susceptibility to downy mildew [16]. Recently, NRR (negative regulator of resistance) was shown to regulate resistance against *Xanthmonas* *oryzae* by cross-talk or overlapping between NH1- and Xa21-mediated pathways [17]. Suppression of *OsSSI2*, which encodes a fatty acid desaturase, enhanced resistance against rice blast disease caused by the fungal pathogen *Magnaporthe* *oryzae* and leaf blight caused by the bacterial pathogen *Xanthmonas* *oryzae* [18]. An enhanced disease-resistance phenotype to both fungal and bacterial pathogens was also observed in the rice *Oscbt-1* mutant, which lacks a calmodulin-binding transcription factor [19].

One strategy to isolate candidates for genes required for susceptibility (plant disease susceptibility factors) is to isolate knockout mutants or create knock-down plants showing a disease-resistant phenotype. Virus-induced gene silencing (VIGS) is a powerful tool for analyzing gene function [20]. Based on this principle, we carried out VIGS screening of genes related to disease susceptibility using *Nicotiana* *benthamiana* and the potato virus X vector system. Previously, we have isolated candidate gene fragments related to disease resistance and susceptibility, designated as *Ralstonia solanacearum* responsive genes from *Nicotiana tabacum* [21]. The *R. solanacearum* responsive genes were randomly cloned into the Ti-PVX vector and transformed into *Agrobacterium tumefaciens*, and then inoculated into *N* *. benthamiana* to create VIGS plants. In this paper, we screened a VIGS plant that barely showed wilting symptoms after inoculation with the pathogen *R. solanacearum*. This VIGS plant was designated as DS1 (Disease suppression 1). In this study, we identified and characterized the DS1 gene. We also analyzed and discussed the molecular mechanisms of the DS1 phenotype.

## Materials and Methods

### Plant Materials

*Nicotiana* *benthamiana* and transgenic *N* *. benthamiana* (NahG) were grown in a growth room under conditions described previously [22].

### Primers and plasmids

The primers and plasmids used in this study are listed in Tables S1 and S2, respectively.

### Microbes, Culture Conditions, and Bacterial Inoculation

*Ralstonia solanacearum* strain OE1-1 (RsOE1-1) and *R. solanacearum* 8107 (Rs8107) were cultured in PY medium containing 20 µg/ml rifampicin, and the *hrp*-deficient mutant of *R. solanacearum* OE1-1 (RsOE1-1ΔY) was cultured in PY medium containing 50 µg/ml kanamycin [22]. Bacterial inoculation was carried out by leaf infiltration as a model experimental system as described elsewhere [23]. The leaf-infiltration method produces the same phenotype in tobacco plants against *R. solanacearum* strains when compared with the root-inoculation method [24,25,26]. Reproducible expression of defense-related genes was also observed in tobacco leaves inoculated with RsOE1-1, Rs8107 and a mutant strain of the bacteria [21,24,25,27].

### Disease Index

The population of RsOE1-1 was determined by plating on Hara-Ono plates. Plants inoculated with RsOE1-1 were coded and inspected daily for wilting symptoms for 14 days. For each plant, a disease index on a scale of 0 to 4 was calculated as described elsewhere [22].

### Isolation of RNA

Total RNA was isolated from *N* *. benthamiana* leaves with RNAiso (Takara Shuzo, Shiga, Japan) according to the manufacturer’s manual. RNA samples were treated with DNase I (RNase-free; Takara Shuzo) to degrade contaminating genomic DNA as described previously [23].

### Isolation of Full-Length cDNA

PCR amplification was performed with the primers DS1Full-S and DS1Full-A. Cycling parameters were as follows: 30 cycles of 94°C for 1 min, 55°C for 1 min, and 72°C for 1 min. The full-length cDNA was cloned into the vector pGEMT-Easy (Promega Co. Ltd., Tokyo, Japan), creating pGEM DS1.

### Sequencing

The PCR products were sequenced using M13 primers M4 and RV with the reagents for the Big Dye Terminator Cycle Sequencing Kit (Applied Biosystems, Foster, CA, USA) and an Applied Biosystems 3100 Avant Automated Sequencer (Applied Biosystems, Warrington, UK) according to the manufacturer’s instructions. The sequence analysis was carried out using DNASIS software (version 3.6; Hitachi, Yokohama, Japan) and the BLAST network service from the National Center for Biotechnology Information. The clustalW program (http://clustalw.ddbj.nig.ac.jp/top-j.html) was used for phylogenic analysis.

### Quantitative Real Time PCR

Quantitative real time polymerase chain reaction (qRT-PCR) was carried out using the method of Maimbo et al. [23]. Reverse transcription was carried out with 1 µg total RNA using PrimeScript RT reagent Kit (Takara). qRT-PCR was carried out in a 20 µl reaction mixture containing 1 µL cDNA stock and 10 pM respective primers using the SYBR GreenER qPCR Reagent System (Invitrogen, Tokyo, Japan), with an Applied Biosystems 7300 real time PCR system (Applied Biosystems). The cycling parameters were the same for all primers: initial 50°C for 2 min and 95°C for 10 min, followed by 40 cycles of 95°C for 10 s and 60°C for 1 min. Melting curve runs were performed at the end of each PCR reaction to verify the specificity of primers by the presence of a single product. The specificity of the primers under these PCR conditions was initially verified by agarose gel electrophoresis that yielded a single product of the expected molecular size. We also checked the sequence of amplified DNA fragments by direct sequencing with an upper primer of each respective gene. Relative quantification of gene expression was carried out according to the instructions for the Applied Biosystems 7300 real-time PCR system, using the comparative cycle threshold [Ct] method to calculate the Qty value. All values were normalized to the expression values of the actin gene as an internal standard in each cDNA stock, as described previously. Expression analyses were carried out with at least two biological replications to ensure that expression patterns were reproducible. Characteristic data are shown in figures. Standard deviations and differences between expression ratios of non-treated controls and other samples were tested for statistical significance using the t-test.

### Semi-quantitative RT-PCR

Reverse transcription-polymerase chain reaction was carried out using Ex-Taq (Takara Shuzo) at denaturing, annealing, and extension temperatures 94°C for 1 min, 56°C for 1 min, and 72°C for 1 min, respectively, for 24 to 26 cycles. The PCR products were separated on 1.0% agarose gels and then stained with ethidium bromide for visualization.

### Vector Constructs and Seedling Infection for Virus-induced Gene Silencing

**Figure 1:**
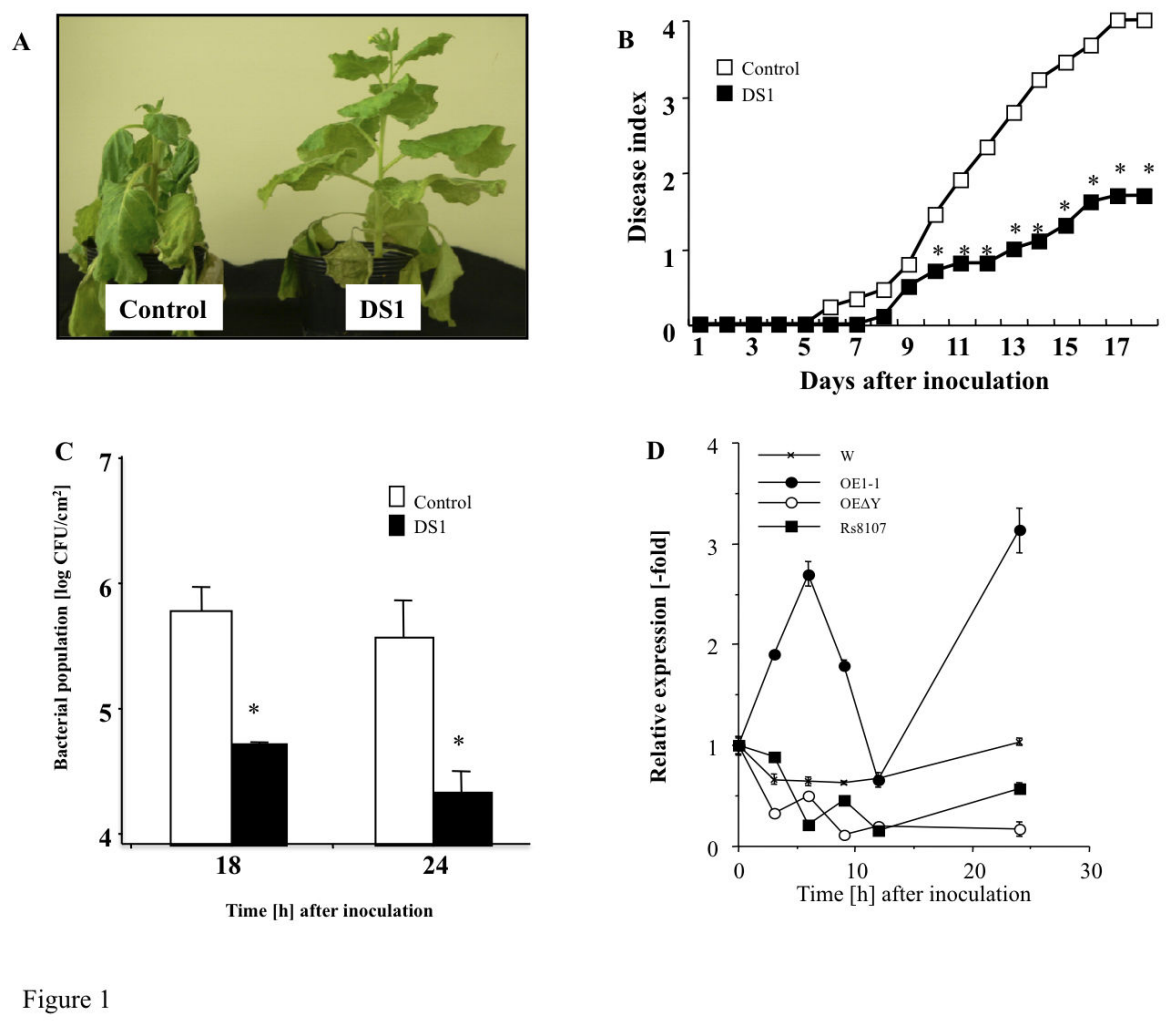
Disease development of bacterial wilt and growth of *Ralstonia solanacearum* in DS1 plants. Control and DS1 plant leaves infiltrated with *R*. *solanacearum*. (*A*) Characteristic symptoms in control and DS1 plants. Photograph was taken 12 days after inoculation with *R*. *solanacearum*. (*B*) Disease development of bacterial wilt was rated daily on a 0–4 disease index in control (open squares) or DS1 (solid squares) plants. Asterisks denote values significantly different from those of control plants (*; *P* < 0.05, *t*-test). (*C*) Control and DS1 plant leaves infiltrated with *R*. *solanacearum* (10^8^ CFU/ml). Bacterial population was determined by plating at specified time points. Values are means of four replicate experiments with SD. Asterisks denote values significantly different from those of empty PVX controls (*; *P* < 0.05, *t*-test). (*D*) Total RNA was isolated from *N*. *benthamiana* leaves inoculated with water, *R*. *solanacearum* strain (RsOE1-1), strain 8107 (Rs8107), and *hrp*-deficient mutant of *R*. *solanacearum* OE1-1 (RsOE1-1ΔY). Transcript levels of DS1 were estimated by qRT-PCR. Values represent mean ± SD from triplicate experiments. Asterisks denote values significantly different from those of water-inoculated controls (*; *P* < 0.05, *t*-test).

A 357-bp cDNA fragment of *DS1* was amplified with primers DS1PVXS and DS1PVXA. This cDNA fragment used for VIGS experiment is shown in Figure 1. This cDNA fragment was subcloned into the TA cloning site of pGEM-T-Easy, creating pGEMDS1PVX. The pGEMDS1PVX plasmid was digested with *Pst*I and *Sal*I, and ligated into the Potato virus X (PVX) vector pPVX201 digested with *Sse*8387I and *Sal*I [20]. The construct containing this insert in the antisense orientation was designated as pPVX:DS1. Silencing vectors for NbrbohB [28] and NbCoi1 [29] were created as reported previously. The plasmid pPVX201 with no insert was used as a control. These binary plasmids were transformed into *A. tumefaciens* strain GV3101, and inoculated into *N* *. benthamiana* leaves as described previously [23]. Three weeks after initial *A. tumefaciens* inoculation, Rs8107, RsOE1-1 and *Agrobacterium* were inoculated into an *N* *. benthamiana* leaf three to four leaves above the *Agrobacterium* -inoculated leaf as a challenge inoculation [23].

#### Plasmid construction and cell-free protein synthesis

The open reading frame of *DS1* fused with a His-tag was amplified and cloned into the wheat germ expression vector pEU3b [30]. The recombinant DS1 was synthesized by a robotic protein synthesizer, Protemist^®^ DT (CellFree Sciences, Yokohama, Japan) according to the manufacturer’s instructions.

### Phosphatidic Acid Phosphatase Activity Assay

Crude protein extract, was prepared as described by Maimbo et al. [22]. The enzyme activity assay was carried out as described by Nakamura et al. [31]. Recombinant protein or crude protein extracts was mixed with substrate solution (45 nCi L-3-PA, 1,2-di[1-14C] palmitoyl, (PerkinElmer, Waltham, MA) and 18 nmol L-3-PA, 1,2-dipalmitoyl dispersed in 50 mM Tris-HCl, pH 7.0, containing 0.1% (wt/vol) Triton X-100 and various concentrations of MgCl_2_) and incubated at 25°C for 1 h. The reaction products were extracted and developed by 1-D TLC (Figure S2B). Radioactive DAG spots were quantified using ImagePlate (Fuji Photofilm, Tokyo, Japan) and Image Analyzer software (Storm, Amersham Biosciences, Tokyo, Japan). Amount of diacylglycerol was calculated by subtracting background counts from counts of diacylglycerol spots.

### Evaluation of Cell Viability

To evaluate viability, leaf discs were stained with Evans blue (Nacalai Tesque, Kyoto, Japan) using the method of Kiba et al. [32].

### Lipid analysis

Leaves were detached from gene-silenced plants, and the leaf petioles were dipped in water containing 0.59 Mbq carrier-free [^32^P] orthophosphate (Muromachi Chemical Co., Tokyo, Japan) and incubated at 25°C. Total lipids were extracted in MeOH:HCl (50:100:1, v/v) using the method of Munnik et al. [33]. Lipid extracts were dried by vacuum centrifugation, dissolved in CHCl_3_, and then separated by thin layer chromatography (TLC). An ethyl acetate solvent system [organic upper phase of ethyl acetate/isooctane/formic acid/H _2_O (13:2:3:10, v/v)] was used to separate PA from other phospholipids [34]. Radiolabeled lipids were visualized by autoradiography, and densitometry scans of autoradiograms were conducted using GE Storm 860 and ImageQuant TL (GE Healthcare, Tokyo, Japan). PA content was calculated by subtracting background counts from counts of PA spots. Characteristic separation pattern of PA is shown in Figure S2B. The PA content was normalized to the dry weight of lipid-extracted leaves.

### Detection of Reactive Oxygen Species

To visualize H_2_O_2_ *in situ*, 3,3′-diaminobenzidine (DAB) staining was performed as described by Maimbo et al. [22]. Inoculated leaves were vacuum-infiltrated with DAB solution, then incubated in the dark until a brown precipitate was observed (approx. 2 h), and then fixed in a solution of 3:1:1 ethanol/lactic acid/glycerol. Quantification of DAB-positive brown spots was carried out with ImageJ software. ROS measurements were conducted as described by Kobayashi et al. [35]. The relative intensity of ROS generation was determined by counting photons from L-012-mediated chemiluminescence. The L-012 probe (0.5 mM in 10 mM MOPS-KOH, pH 7.4) was infiltrated into *N* *. benthamiana* leaves using a needleless syringe. Chemiluminescence was monitored continuously using a photon image processor equipped with a sensitive CCD camera (LAS-4000 mini) with Multi Gauge ver. 3.0 software (Fujifilm, Tokyo, Japan).

### Creation of Transgenic Tobacco Plants

We created a binary vector containing the *DS1* gene expressed under the control of the 35S promoter. The full-length open reading frame of *DS1* was amplified with the primers DS1ox-S and DS1ox-A using pGEMDS1 as the template and cloned into pGEMT-Easy, yielding pGEMDS1-2. pGEMDS1-2 was digested with BamHI and *Sac*I (Takara Bio), and ligated into the pBI121 vector (Clontech, Tokyo, Japan) digested with the same enzymes. The construct containing the insert was designated as pBI-DS1. Tobacco plants (*N. tabacum* cv. Samsun NN) aseptically grown from seed for approximately 1 month were transformed with *DS1* via the *A. tumefaciens*-mediated leaf disc procedure [36] and selected using 5 µg ml^-1^ kanamycin as the selection reagent. Successful transformation with the *DS1* gene was confirmed by genomic PCR analysis of DS1-OX#2 and DS1-OX#8 plants with primers based on CaMV35S promoter (35S-F) and NOS terminator (Nos-R) sequences.

## Results

### DS1 plants show strong resistance to *R. solanacearum*

We screened a gene knock-down plant showing significant resistance to *R. solanacearum* by high throughput VIGS, and designated the plant as DS1 (Disease suppression 1) (Figure 1A, B). The DS1 plant showed an enhanced-resistance phenotype, because the bacterial population was significantly reduced in the DS1 plant compared with that in controls (Figure 1C). There was no noticeable morphological difference between control and DS1 plants (Figure S3A).

To examine the expression profile of DS1 during bacterial infection, RNA samples were isolated from *N* *. benthamiana* leaves inoculated with a virulent strain of *R. solanacearum* (RsOE1-1), an avirulent strain of *R. solanacearum* (Rs8107), and the corresponding *hrpY* (encoding Hrp pilus)-mutant of RsOE1-1 (RsOE1-1ΔY). A drastic increase in *DS1* transcript abundance was observed in RsOE1-1-inoculated *N* *. benthamiana* , with peak levels of *DS1* transcripts in tobacco at 12 h and 24 h after inoculation. In contrast, the transcript abundance of *DS1* was not affected by inoculation with Rs8107 and RsOE1-1ΔY 6 HAI (Figure 1D). These results suggest that *DS1* was induced in response to infection with a virulent strain of *R. solanacearum* in a type III secretion system-dependent manner.

### DS1 is a phosphatidic acid phosphatase

**Figure 2:**
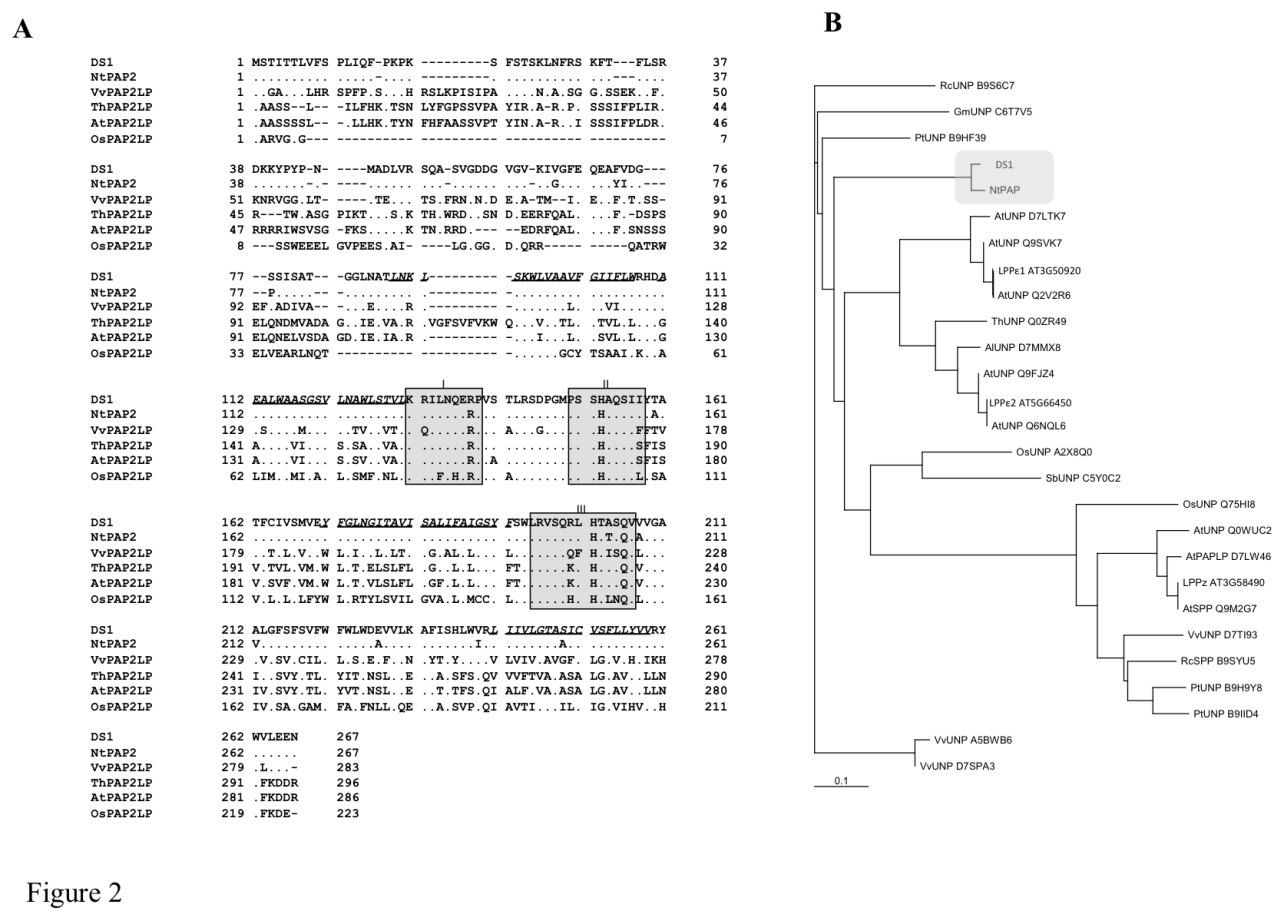
Deduced amino acid sequence and phylogenic analysis of DS1. (*A*) Alignment of deduced amino acid sequence of *DS1* and its ortholog in *N*. *tabacum* (NtPAP), phosphatidic acid phosphatase 2-like proteins from *Vitis* *vinifera* (VvPAPLP), *Thellungiella* *halophila* (ThPAP2LP), *Arabidopsis* *thaliana* (AtPAP2LP), and *Oryza* *sativa* (OsPAP2LP). Dots show identical amino acids, bars show amino acids that are not present in sequences. Boxes show conserved PAP catalytic motifs I, II, and III essential for PAP activity. Putative transmembrane motifs are shown in italic with underline. (B) Phylogenic tree of lipid phosphatases in plants. Gray boxes show DS1 and its ortholog in *N*. *tabacum*. The scale bar represents 0.1 JTT distance matrix units.

**Figure 3:**
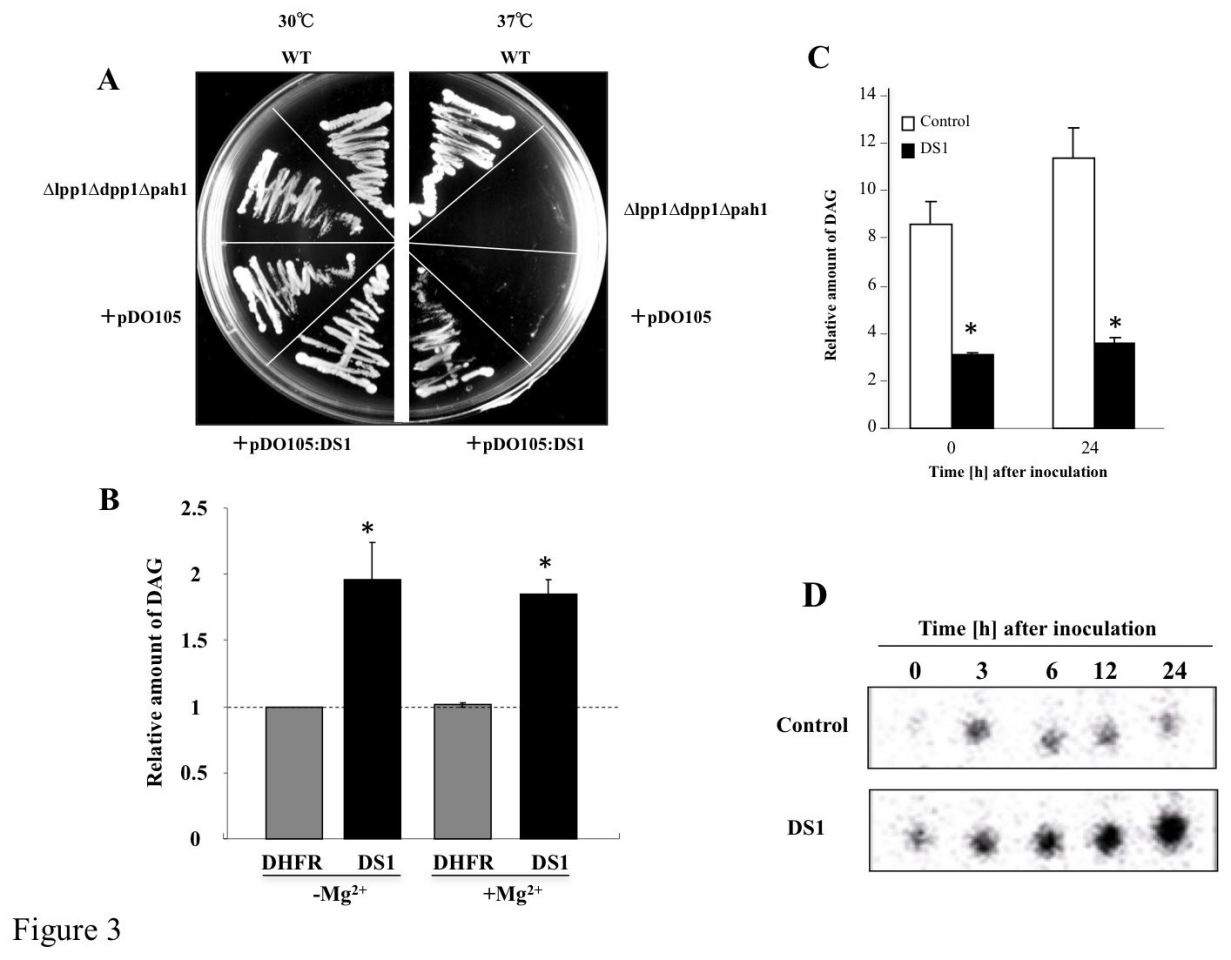
Functional analysis of DS1. (*A*) Isogenic yeast strain ∆lpp1∆dpp1∆pah1 containing empty pDO105 plasmid (+pDO105) or pDO105 containing DS1 (+pDO105: DS1) were cultured on agar YPD plates and incubated at 30°C or 37°C. (*B*) PAP activity of DS1 and dehydrofolate reductase (DHFR; negative control) was determined in the presence or absence of Mg^2+^ as described in Materials and Methods. Values are means and SD from triplicate experiments. Asterisks denote values significantly different from those of control (*; *P* < 0.05). (*C*) PAP activity in control (white bar) and DS1 plants (black bar). Crude protein fractions were isolated from control and DS1 plants 0 and 24 h after inoculation with *R*. *solanacearum*. PAP activity was determined without Mg^2+^ as described in Materials and Methods. Values are means and SD from triplicate experiments. Asterisks denote values significantly different from those of control (*; *P* < 0.05). (*D*) Phosphatidic acid contents in control and DS1 plants. Total lipid fraction was extracted from control and DS1 plants 0 to 24 h after inoculation with *R*. *solanacearum*. PA was separated by ethyl acetate TLC as described in Materials and Methods.

The full-length cDNA corresponding to the phenotype for DS1 plants contained an open reading frame encoding a polypeptide of 268 amino acids (Accession No. AB818894). The predicted molecular mass was calculated as approximately 29.6 kDa. A protein database search showed 95.8% amino acid identity with its ortholog in *N. tabacum*, and 34.7%, 30.9%, 30.0%, and 28.3% amino acid identity with putative phosphatidic acid phosphatase (PAP) 2 proteins from *Vitis vinifera*, *Thellungiella* *halophila* , *Oryza sativa*, and *Arabidopsis thaliana*, respectively (Figure 2A). Phylogenic analysis identified at least 8 groups of lipid phosphatases, and DS1 and its ortholog in *N. tabacum* were classified into independent cluster adjacent to the cluster including the prokaryotic PAP, such as AtLPPε1 and 2 (Figure 2B; Nakamura et al. 2009). The deduced amino acid sequence contained catalytic motifs I, II, and III [37,38], including R135, H153, and H202 [39,40], which are essential for phosphatidic acid phosphatase activity (Figure 2A). Introduction of *DS1* into the yeast *∆lpp1∆dpp1∆pah1* mutant, a temperature-sensitive PAP mutant of yeast, rescued growth defects at 37°C (Figure 3A). Furthermore, the recombinant DS1 protein hydrolyzed phosphatidic acid (PA) in an Mg^2+^-independent manner (Figure 3B). These results indicate that DS1 has PAP activity with the characteristic features of a PAP2.

Because the DS1 protein showed PAP activity in yeast cells and *in vitro*, we expected that PAP activity would be altered in DS1 plants. We analyzed PAP activity in crude protein fractions from control and DS1 plants. We detected PAP activity in control plants without inoculation, and PAP activity increased in 0 time control plants at 24 h after inoculation with RsOE1-1. In contrast, PAP drastically decreased in DS1 plants at 0 time control and 24 h after inoculation with RsOE1-1 (Figure 3C). Since there was decreased PAP activity in DS1 plants, we expected that the PA content would differ between DS1 and control plants. Comparison of PA content between DS1 plants and control plants showed an approximately 2-fold accumulation of PA in DS1 plants without challenge inoculation, compared with that in control plants. PA accumulation drastically increased in DS1 plants inoculated with RsOE1-1 (Figure 3D) at 24 h after inoculation with RsOE1-1, DS1 plants showed approximately 200-fold accumulation of PA, compared with that in control plants. These results indicate that the DS1 protein had PAP activity *in planta*, and abnormal accumulation of PA in DS1 plants was due to reduced PAP activity.

### DS1 plants show rapid activation of immune responses during the infection with *R. solanacearum*

**Figure 4:**
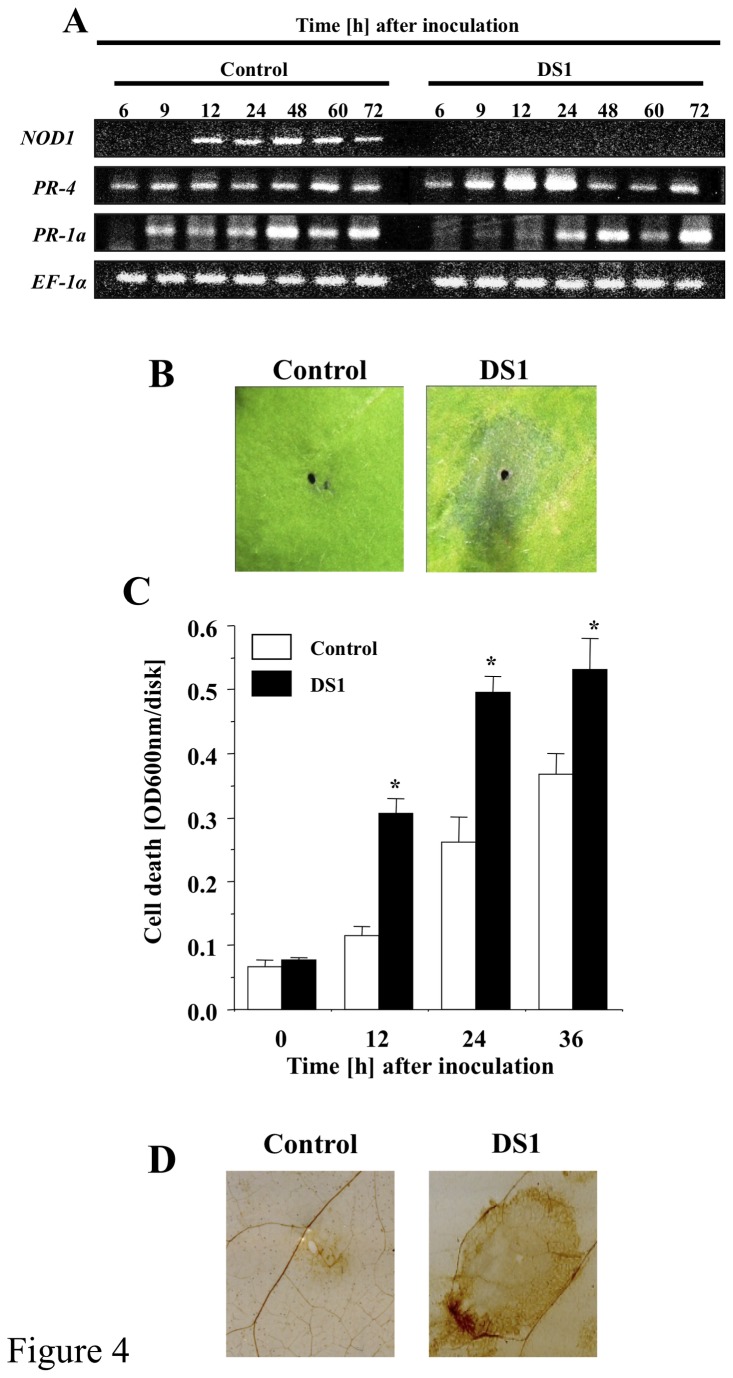
Up-regulation of defense-related responses in DS1 plants against *R. solanacearum* infection. (*A*) Total RNA was isolated from control (Control) and DS1 plants (DS1) 6 to 72 h after inoculation with *R*. *solanacearum*. Expression values of *DS1*, *PR-1a*, *PR-4*, and *NbEF-1α* were analyzed by semi-quantitative RT-PCR with specific primers for *NbPR-1a*, *NbPR-4*, *DS1*, and *NbEF-1α*. (*B*) HR-like lesion formation in DS1 plant in response to *R*. *solanacearum* infection. Control and DS1 plants were infiltrated with *R*. *solanacearum*. Pictures of *N*. *benthamiana* leaves taken 36 hours after bacterial infiltration. (*C*) Cell death was determined by Evans blue staining. Values are means of four replicate experiments with SD. Asterisks denote values significantly different from those of empty PVX control (*; *P* < 0.05, *t*-test). (*D*) ROS production was visualized 48 h after inoculation with *R*. *solanacearum* by DAB staining as described in Materials and Methods.

Because elevated resistance to pathogens usually correlates with induction of defense responses, we analyzed characteristic defense responses in control and DS1 plants challenged with RsOE1-1. The DS1 plants showed elevated expression of *PR-4*, a marker gene for the jasmonic acid-dependent (JA-dependent) signaling pathway, at 12 and 24 h after inoculation with RsOE1-1. In contrast, expression of the *PR-1a* gene, a marker gene for the salicylic acid-dependent (SA-dependent) signaling pathway, was not enhanced, but suppressed, in DS1 plants 6 to 12 h after inoculation with RsOE1-1 (Figure 4A). The DS1 plants showed accelerated lesion formation (hypersensitive response-like lesions) in response to RsOE1-1 infiltration (Figure 4B). Induction of cell death also accelerated in DS1 plants (Figure 4C). Plants undergoing cell death usually produce reactive oxygen species (ROS). Therefore, we evaluated ROS in control and DS1 plants in response to RsOE1-1 infection. Staining of H_2_O_2_ with DAB staining revealed numerous brown patches on DS1 plants, which were comparable to those on control plants infected with RsOE1-1 (Figure 4D). However, we did not observe such defense-related responses in either control or DS1 plants without RsOE1-1 infection (Figure S3B,C,D). These results indicate that DS1 plants did not show the hallmarks of a constitutive defense response, but instead displayed enhanced responsiveness to bacterial infection.

### *DS1*-overexpressing transgenic tobacco plants are highly susceptible to *R. solanacearum*

A reduction of *DS1* gene expression by VIGS caused over-accumulation of PA, resulting in enhanced resistance to RsOE1-1. This suggests that immune responses might be down-regulated via degradation of PA by DS1-PAP activity. These results prompted us to determine whether overexpression of *DS1* reduced immune responses to RsOE1-1. We created *DS1*-overexpressing transgenic tobacco plants. Successful transformation with the *DS1* gene was also confirmed by genomic PCR analysis of DS1-OX#2 and DS1-OX#8 plants (Figure S4A). To select *DS1*-overexpressing lines, we first compared the total expression levels of *DS1* among transgenic plants. Two transgenic T_3_ plant lines homozygous for kanamycin resistance, DS1-OX#2 and DS1-OX#8, showed elevated levels of total *DS1* expression compared with that in control plants (Figure S4B). There was no noticeable morphological difference between control and DS1-OX#2/ DS1-OX#8 plants (Figure S4C).

**Figure 5:**
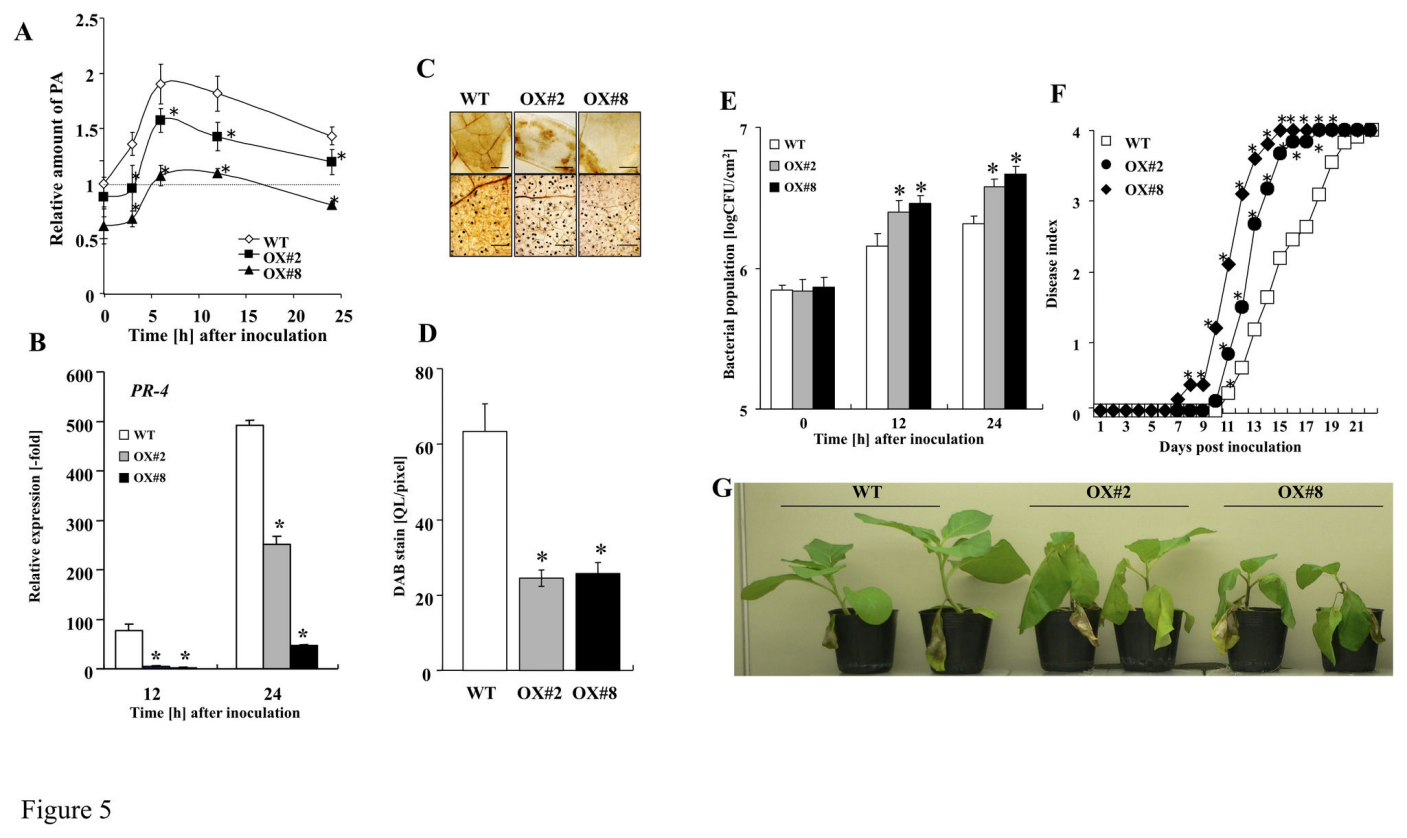
Reduction of defense-related responses and disease resistance in DS1-overexpressing plants. (*A*) Phosphatidic acid content in control and *DS1*-overexpressing plants (OX-#2 and OX-#8). Total lipid fraction was extracted from control (WT) and *DS1*-overexpressing plants (OX-#2 and OX-#8) 0–24 h after inoculation with *R*. *solanacearum*. PA was separated and quantified as described in Materials and Methods. Values are means of four replicate experiments with SD. Asterisks denote values significantly different from those of control (*; *P* < 0.05, t-test). (*B*) Total RNA was isolated from wild-type control (Control), *DS1*-overexpressing plants (OX-#2 and OX-#8) at 12 and 24 h after inoculation with *R*. *solanacearum*. Expression values of *PR-4* are expressed as [Qty] after normalization to actin. Values represent means and SD from triplicate experiments. Asterisks denote values significantly different from those of control (*; *P* < 0.05, *t*-test). (*C*) ROS production was visualized by DAB staining as described in Materials and Methods. Bar indicates @@@. (*D*) Brown deposits were quantified as described in Materials and Methods. (*E*) Bacterial population was determined by plating at specified time points. Values are means of four replicate experiments with SD. Asterisks denote values significantly different from those of control (*; *P* < 0.05, *t*-test). (*F*) Disease development of bacterial wilt was rated daily on a 0–4 disease index in control (WT) and *DS1*-overexpressing (OX#2 and OX#8) plants. Values are means of four replicate experiments with SD. Asterisks denote values significantly different from those of empty PVX control (*; *P* < 0.05, t-test). (*G*) Characteristic symptoms in control and *DS1*-overexpressing (OX#2 and OX#8) plants. Photograph was taken 12 days after inoculation with *R*. *solanacearum*.

In DS1-OX#2 and DS1-OX#8 plants, PA content was significantly reduced compared with that in control plants with or without RsOE1-1 inoculation (Figure 5A). The expression level of *PR-4* decreased in DS1-OX#2 and DS1-OX#8 plants challenged with RsOE1-1 (Figure 5B). ROS production also decreased in DS1-OX#2 and DS1-OX#8 plants challenged with RsOE1-1 (Figure 5C,D), and bacterial growth of RsOE1-1 was significantly enhanced compared with that in control plants (Figure 5E). The appearance of wilt symptoms also accelerated in DS1-OX#2 and DS1-OX#8 plants (Figure 5F,G). These results indicate that *DS1*-overexpression induced susceptibility to RsOE1-1 via degradation of PA.

### rbohB-dependent reactive oxygen has a role in DS1 phenotype

**Figure 6:**
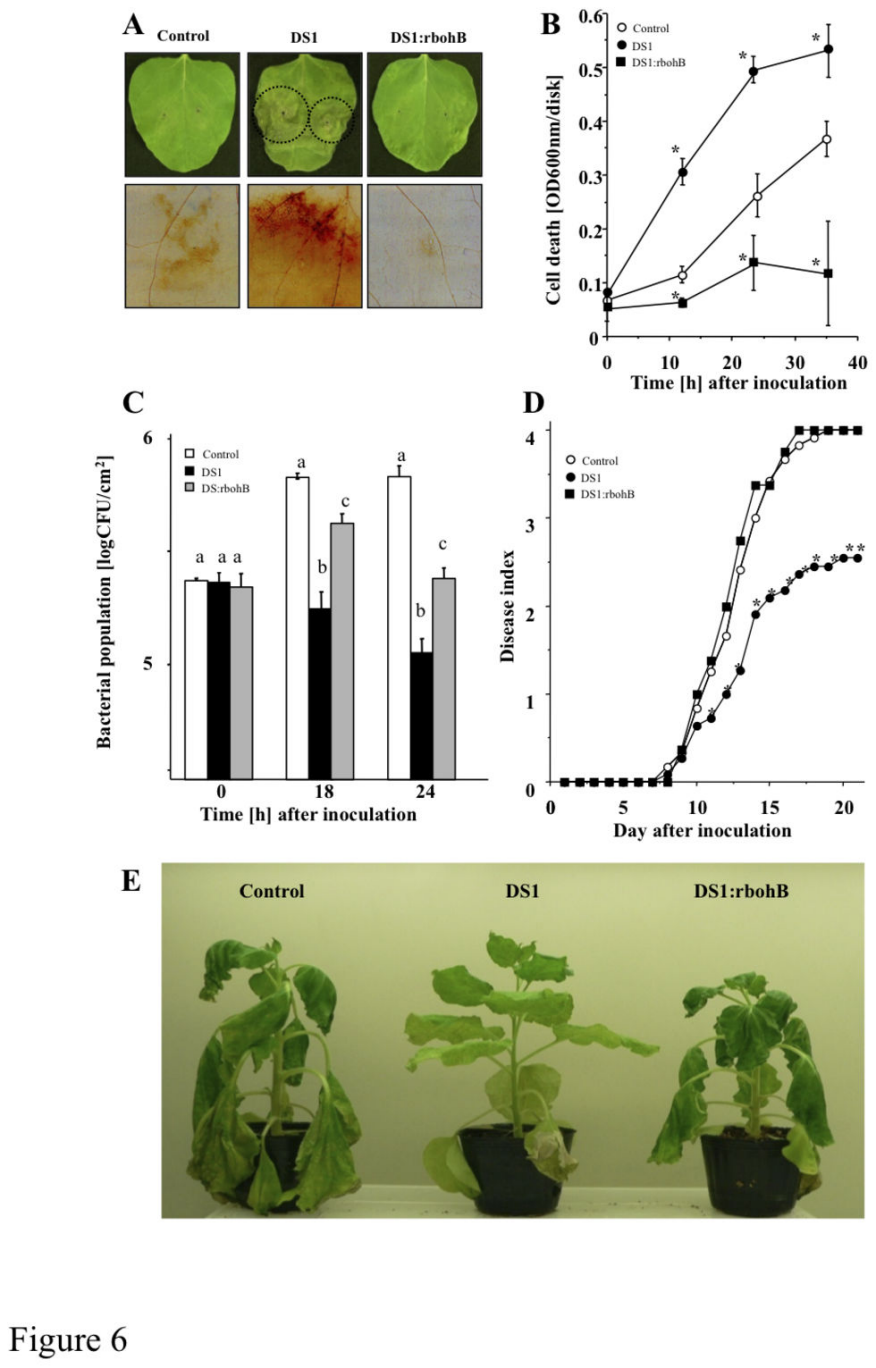
Role of NbrboHB in DS1 phenotype Control, DS1, and DS1:NbrboHB (DS1:rbohB) double-knockdown plant leaves were infiltrated with *R. solanacearum*. (*A*) Photograph of HR-like lesion was taken 36 h after inoculation Dot lined circles showed lesion developed areas. ROS production was visualized 48 h after inoculation by DAB staining as described in Materials and Methods. Bars indicated 500 nm. (*B*) Cell death was determined by Evans blue staining (OD_600_ disk^-1^, see Materials and Methods). (*C*) Bacterial population was determined by plating at specified time points. Values are means of four replicate experiments with SD. Different letters show significant differences among control, DS1, and double-knockdown plants (ANOVA test). (*D*) Disease development of bacterial wilt was rated daily on a 0–4 disease index in control (open circles), DS1 (solid circles), or DS1:NbrboHB (closed squares) plants. Values are means of four replicate experiments with SD. Different letters show significant differences among controls, DS1, and double-knockdown plants (p<0.05 ANOVA). (*E*) Characteristic symptoms in control, DS1, and DS1:rboHB plants. Photograph was taken 12 days after inoculation with *R. solanacearum*.

DS1 plants showed increased ROS accumulation in response to RsOE1-1 infection. Previous reports show that elevated ROS levels are sufficient to enhance plant immune responses. A major source of ROS is the membrane-bound NADPH oxidase, NbrbohB, in *N* *. benthamiana* [22]. To determine if the reduced growth of RsOE1-1 and the DS1 phenotype were caused by NbrbohB-dependent accumulation of ROS, we created plants in which both *DS1* and *NbrbohB* were silenced (DS1:rboHB plants; Figures S5, 6). In DS1:rboHB plants, elevated ROS production was compromised in response to RsOE1-1 infection, and the level of ROS accumulation was lower than that in control plants (Figure 6A). Intriguingly, the accelerated-cell-death phenotype in response to RsOE1-1 infiltration was also compromised in DS1:rboHB plants (Figure 6B). The enhanced-resistance phenotype was weaker in DS1:rboHB plants than in DS1 plants, since there was greater bacterial growth in DS1:rboHB plants than in DS1 plants (Figure 6C). Consistent with bacterial growth, DS1:rboHB plants showed wilt symptoms (Figure 6D,E). These results indicate that elevated ROS production via NbrbohB is involved in the DS1 phenotype.

### Role of jasmonic acid pathway in DS1 phenotype

**Figure 7:**
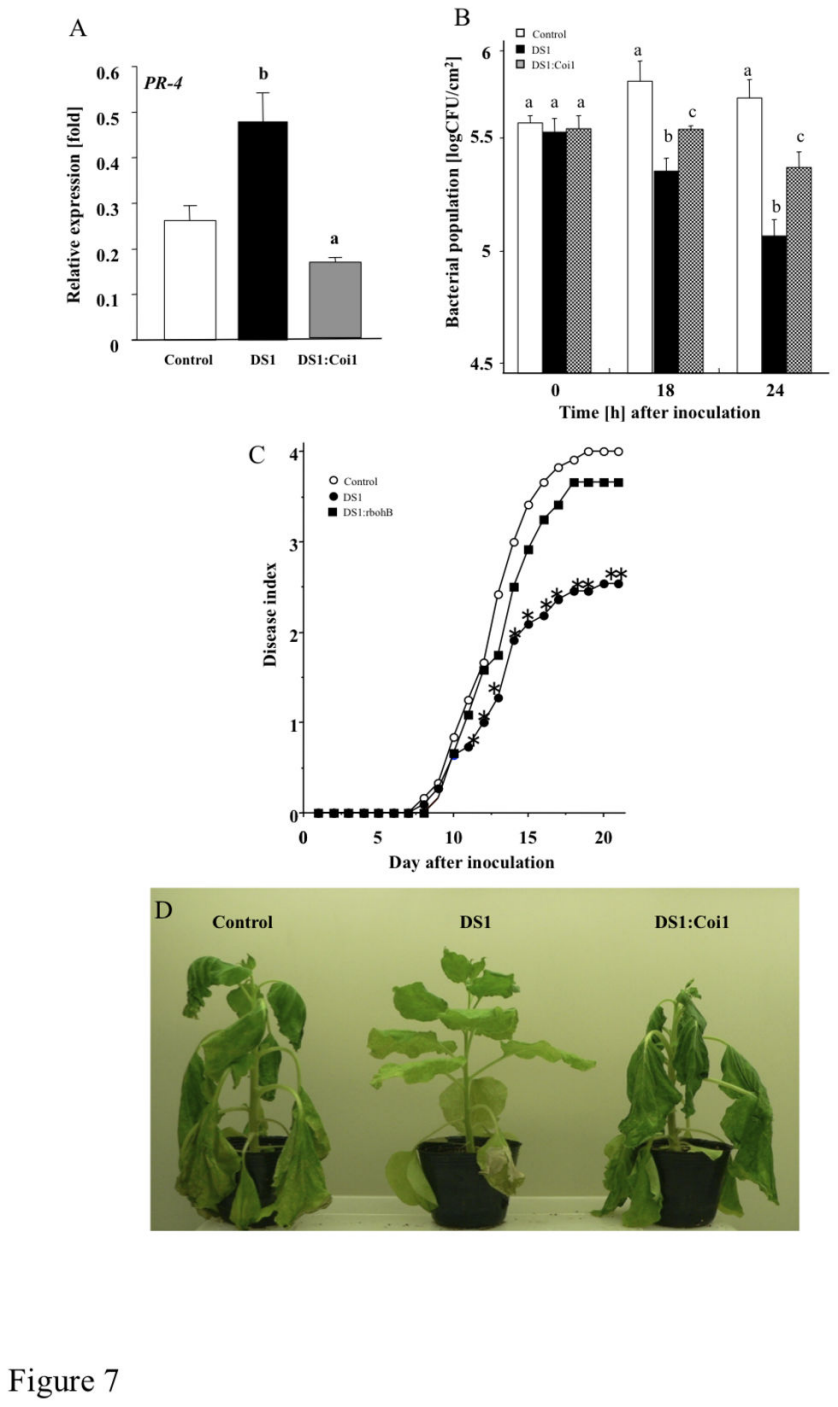
Role of jasmonic acid pathway in DS1 phenotype. Control, DS1, and DS1:Coi1 double-knockdown plant leaves were infiltrated with *R*. *solanacearum*. (*A*) Total RNA was isolated from control (Control), DS1 (DS1), and DS1:NbCoi1 (DS1:Coi1) double-knockdown plant 24 h after inoculation with *R*. *solanacearum*. Expression values of *PR-4* are expressed as [Qty] after normalization to actin. Values represent means and SD from triplicate experiments. Different letters show significant differences among control, DS1, and double-knockdown plants (ANOVA test). (*B*) Bacterial population was determined by plating at specified time points. Values are means of four replicate experiments with SD. Different letters show significant differences among controls, DS1, and double-knockdown plants (*p*<0.05 ANOVA). (*C*) Disease development of bacterial wilt was rated daily on a 0–4 disease index in control (open circles), DS1 (solid circles), or DS1:Coi1 (closed squares) plants. Values are means of four replicate experiments with SD. Asterisks denote values significantly different from those of empty PVX control (*; *P* < 0.05, *t*-test). (*D*) Characteristic symptoms in control, DS1, and DS1:Coi1 plants. Photograph was taken 12 days after inoculation with *R*. *solanacearum*.

Since DS1 plants showed rapid activation of JA-dependent *PR-4* expression in response to RsOE1-1 infection, we further analyzed the role of the JA pathway in the DS1 phenotype. We created plants with knocked-down *NbCoi1*, which encodes an F-box protein required for JA signaling, from DS1 plants (DS1:Coi1 plant; Figures S5, 6). zDS1:Coi1 plants showed reduced *PR-4* expression 24 h after inoculation with RsOE1-1 (Figure 7A). We observed partial reduction of the DS1 phenotype in DS1:Coi1 plants, since bacterial growth was greater in DS1:Coi1 plants than in DS1 plants (Figure 7B). DS1:Coi1 plants also showed stronger wilt symptoms than DS1 plants (Figure 7C,D). Plants expressing a salicylic acid-degrading (SA-degrading) enzyme (NahG plants) showed a DS1-like phenotype, similar to that of DS1 plants (Figure S7).

## Discussion

In plant–bacterial pathogen interactions, pathogens suppress plant innate immunity using effectors secreted through type III secretion systems. Some bacterial effectors suppress plant innate immunity by activating effector targets that function as negative regulators of the plant immunity system. For example, *Os8N3*, a genetically dominant rice gene that is up-regulated by a bacterial type-III effector protein, confers gene-for-gene-specified disease susceptibility [41]. We found that *DS1* was up-regulated by infection with a compatible RsOE1-1, but not by incompatible Rs8107. Intriguingly, induction of DS1 required the type III secretion system of RsOE1-1, suggesting that DS1 was induced by type III effector(s) secreted from RsOE1-1 during the infection process (Figure 1D). The data presented here suggests that DS1 may be a target molecule for type III effector(s), and may function as a signaling component in disease susceptibility responses during plant–pathogen interactions. Therefore, identification of type III effector(s) that target DS1 will provide novel insights into how RsOE1-1 suppresses plant immune responses.

From the results of this study, we found that *DS1* encoded PAP (EC 3.1.3.4) (Figure 2), which dephosphorylates PA to yield DAG and inorganic phosphate. PAPs are categorized into either PAP1 or PAP2 based on their enzymatic properties. PAP1 is primarily a soluble enzyme [42], its activity requires Mg^2+^, and it is considered to play roles in lipid metabolism [43]. PAP2 is an integral membrane protein with Mg^2+^-independent activity [38]. We could find a transmembrane motif in a deduced amino acid sequence of DS1 (Figure 2A). In addition, we found that DS1 is classified into independent cluster adjacent to the cluster including the prokaryotic PAP, such as AtLPPε1 and 2 (Figure 2B), and DS1 is localized in chloroplast (Figure S8). Recombinant *DS1* protein showed Mg^2+^-independent PAP activity (Figure 3B). These results suggest that *DS1* encodes PAP2. PAP2 is considered to have a signaling function [44]. For example, expression of a PAP2-type lipid phosphate phosphatase (LPPα1) was induced transiently by radiating gamma rays, ultraviolet-B radiation, or eliciting with mastoparan or harpin [45]. Another member of the PAP2 family, LPPα2, plays an important role in abscisic acid signaling during seed germination [46]. In these experiments, we observed induction of the *DS1* gene in response to RsOE1-1 infection. In addition, *DS1* plants showed elevated resistance to RsOE1-1 (Figure 1A,B,C). Taken together, these results suggest that DS1 might have a role in regulation of signal transduction during immune responses.

The appearance of the DS1 phenotype correlated well with PA accumulation, since DS1 plants over-accumulated PA during RsOE1-1 infection, and *DS1*-overexpressing plants with significantly reduced PA content were more susceptible to the bacteria (Figures 3D, 5). These results suggest that PA might be a critical component of the DS1 phenotype. PA has been implicated in numerous stress responses of plants, and intracellular PA levels increase under various biotic and abiotic stress conditions, including pathogen elicitation [47,48], wounding [49,50], and hyperosmotic stress [51]. It has been suggested that PA is a second messenger in a diverse range of stress-signaling pathways in plants and mediates important stress responses. PA regulates a wide range of important cellular processes in plants, including regulation of ROS generation [52], MAPK activity [53], K^+^-channels [54], and jasmonic acid signaling [55]. Wound-induced PA accumulation causes JA accumulation in *Arabidopsis* plants [56]. A recent study shows that PA binds to NADPH oxidase, RbohD, and RbohF, and activates ROS generation in *Arabidopsis* [57]. Another report shows that PA interacts with Constitutive triple response 1 (CTR1) protein kinase, and promotes ET responses [58]. Recently, it was reported that PA directly binds to Ca^2+^-dependent protein kinases, promoting the activities of these enzymes to activate intracellular signaling [59]. Previously, we reported that the NbSEC14 lipid transfer protein affected phospholipase activity and PA content, leading to plant immune responses in response to pathogen infection [60]. The results of the present study suggests that the DS1 phenotype was due to activation of rboHB-dependent ROS production and Coi1-regulated JA-dependent immune responses, since enhanced resistance decreased in the double-knock down of *DS1* and *NbrbohB* or *NbCoil* (Figures 6, 7). In contrast, another plant hormonal substance, salicylic acid, which is well known to be related to plant innumity, might not be associated to DS1 phenotype, since silencing of *DS1* in NahG plants did not inhibit the DS1 phenotype (Figure S7). Taken together, these results indicate that over-accumulation of PA may stimulate defense-related intracellular signaling cascades via JA and ROS pathways in DS1 plants during infection with RsOE1-1, and that the DS1 protein might negatively regulate plant immune responses via degradation of PA in *N* *. benthamiana* .

In conclusion, interference or removal of negative regulators of defense responses is an effective approach in activating plant immune responses [61]. Therefore, DS1 might be a novel target for plant protection practice, and direct suppression of DS1 function or artificial regulation of signaling cascade upstream of the *DS1* gene could produce quick activation of plant defense to achieve effective resistance. Furthermore, confirmation is needed of the functional link between DS1, PA accumulation and activation of plant immune responses.

## Supporting Information

Figure S1 **Nucleotide sequence of DS1.** Nucleotide sequence of DS1. cDNA fragments used for VIGS experiments are shown with asteriscks. Primer positions are indicated as arrows.
(TIFF)

Figure S2 **Separation and identification of phospholipids by TLC.** Separation and identification of ^32^P-labeled PA (A) or C14-labeled diacylglycerol (B) by TLC. The migration of authentic phospholipid standards are indicated to the right of the TLC plate.
(TIFF)

Figure S3 **Phenotypic observation of DS1 plant.** (A) Photograph was taken 3 weeks after inoculation with *Agrobacterium* *tumefaciens*. Values represent mean plant length (*n* = 7) with SD. (B) ROS production was determined by chemiluminescence as described in Materials and methods. (C) Cell death was determined by Evans blue staining. (D) Total RNA was isolated from control (Control) and DS1 plants (DS1). Transcript levels of DS1 were estimated by qRT-PCR. Values represent mean ± SD from triplicate experiments.
(TIFF)

Figure S4 ***DS1*-overexpressing transgenic tobacco.** (A) Detection of *DS1* gene in transgenic tobacco genome. Total genomic DNA was prepared from wild-type control (WT) or *DS1*-transformed plants (OX#2 and 8). Transformed DS1 was detected by RT-PCR using 35S promoter primer and NOS terminator primer. (B) Expression of DS1 in transgenic tobacco plants. Total RNA was isolated from fully expanded tobacco leaves of wild-type control (WT) or *DS1*-transformed plants (OX#2 and 8). Relative expression value of *DS1* transcripts is shown to relative to that in wild-type control. Values are means of four replicate experiments with SD. Asterisks denote values significantly different from those of controls (*; *P* < 0.05, *t*-test). (C) Morphological observation of *DS1*-overexpressing plant. Photograph was taken 2 months after germination.
(TIFF)

Figure S5 **Phenotypic observation of NbrbohB and NbCoi1-silenced plants.** (A) Total RNA was isolated from control, control, rboHB, and Coi1 plants 0, 12 and 24 h after inoculation with *R. solanacearum*. Semi quantitative RT-PCR was carried out with specific primers for *NbCoi1* and *NbrboHB*. Equal loads of cDNA were monitored by amplifying constitutively expressed *NbEF-1α*. (B) Control and respective silenced plant leaves infiltrated with *R. solanacearum* (10^8^ CFU/ml). Bacterial population was determined by plating at specified time points. Values are means of four replicate experiments with SD. Asterisks denote values significantly different from those of empty PVX controls (*; *P* < 0.05, *t*-test).
(TIFF)

Figure S6 **Phenotypic observation of double knock-down plants.** (A) Total RNA was isolated from control, DS1, DS1:rboHB, and DS1:Coi1 plants 0–24 h after inoculation with *R. solanacearum*. Semi quantitative RT-PCR was carried out with specific primers for *NbCoi1*, *NbrboHB*, and *DS1*. Equal loads of cDNA were monitored by amplifying constitutively expressed *NbEF-1α*. (B) Control, DS1, DS1:rboHB, and DS1:Coi1 plants were photographed 3 weeks after inoculation with *Agrobacterium* *tumefaciens.*
(TIFF)

Figure S7 **Role of salicylic acid in DS1 phenotype.** (A) Total RNA was isolated from NahG and *DS1*-silenced NahG (DS1-NahG) plants 0–24 h after inoculation with *R. solanacearum*. Semi quantitative RT-PCR was carried out with specific primers for DS1. Equal loads of cDNA were monitored by amplifying constitutively expressed *NbEF-1α*. (B) Bacterial population was determined by plating at specified time points. Values are means of four replicate experiments with SD. Asterisks denote values significantly different from those of control (*; *P* < 0.05, *t*-test). (C) Disease development of bacterial wilt was rated daily on a 0–4 disease index in wild-type *N. benthamiana* control (Control), DS1 (DS1), NahG-control (NahG-Control), and NahG- DS1 (NahG-DS1) plants. Values are means of four replicate experiments with SD. Asterisks denote values significantly different from those of empty PVX control (*; *P* < 0.05, *t*-test). (D) Characteristic symptoms in control, DS1, NahG, and NahG-DS1 plants. Photograph was taken 12 days after inoculation with *R. solanacearum*.
(TIFF)

Figure S8 **Subcellular localization of DS1.** Localization of DS1-GFP in *Nicotiana* *benthamiana* protoplasts. Confocal images of protoplasts prepared from leaves inoculated with *Agrobacterium* *tumefaciens* carrying P35S-GFP (GFP) or p35S-DS1-GFP for 48 h. Observation of GFP fluorescence was carried out using the method described previously [62]. Scale bar represents 20 μm.
(TIFF)

Table S1 **List of primers used in this study.**
(TIFF)

Table S2 **List of plasmids used in this study.**
(TIFF)
