# Supplementary figures and images for: Suppression of DS1 Phosphatidic Acid Phosphatase Confirms Resistance to Ralstonia solanacearum in Nicotiana benthamiana
Source: PLoS One. 2013 Sep 20;8(9):e75124. doi: 10.1371/journal.pone.0075124 (PMC3779229; doi:10.1371/journal.pone.0075124)

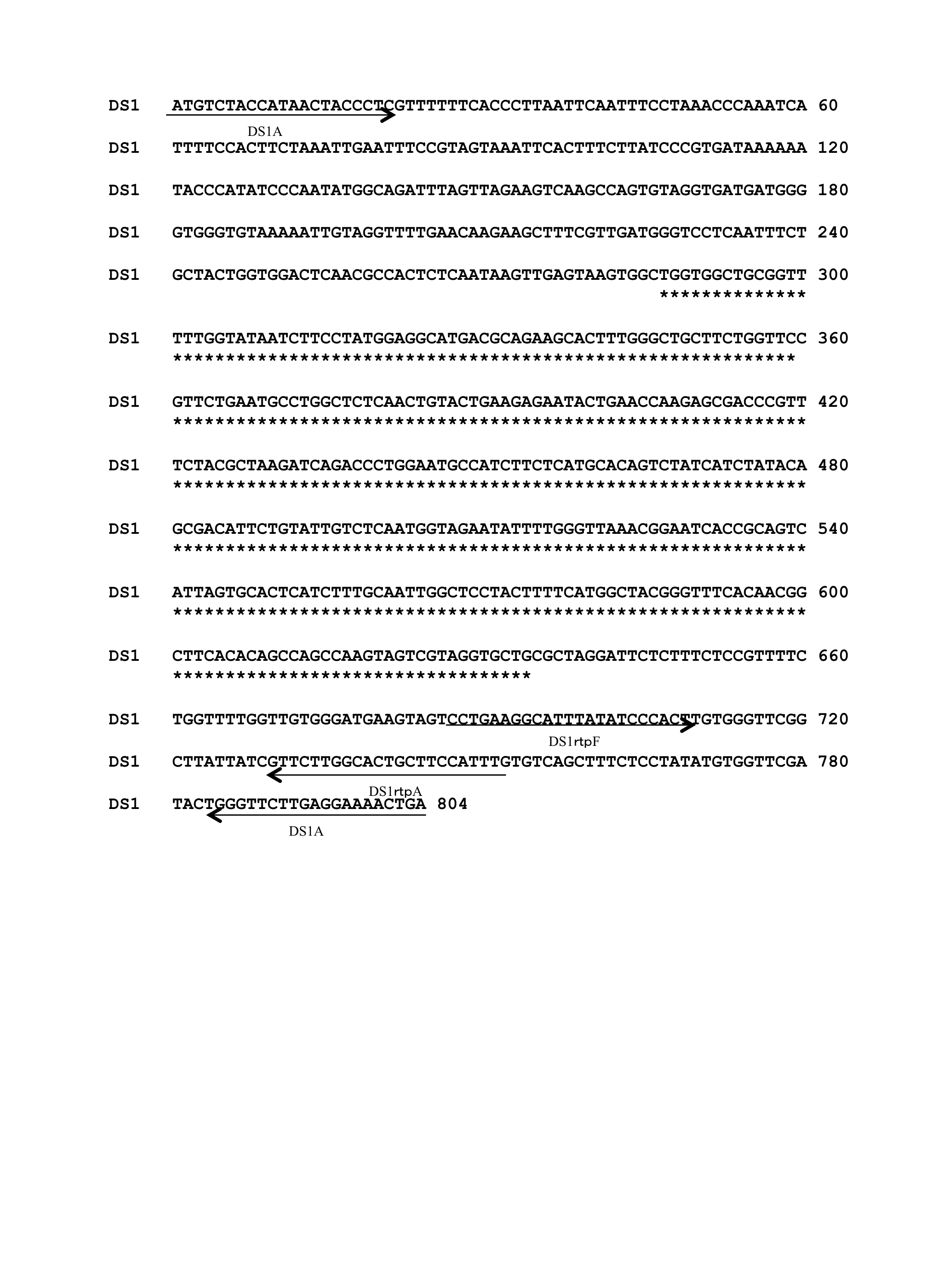

Supplement: Figure S1 — Nucleotide sequence of DS1. Nucleotide sequence of DS1. cDNA fragments used for VIGS experiments are shown with asteriscks. Primer positions are indicated as arrows. (TIFF) [file pone.0075124.s001.tiff]

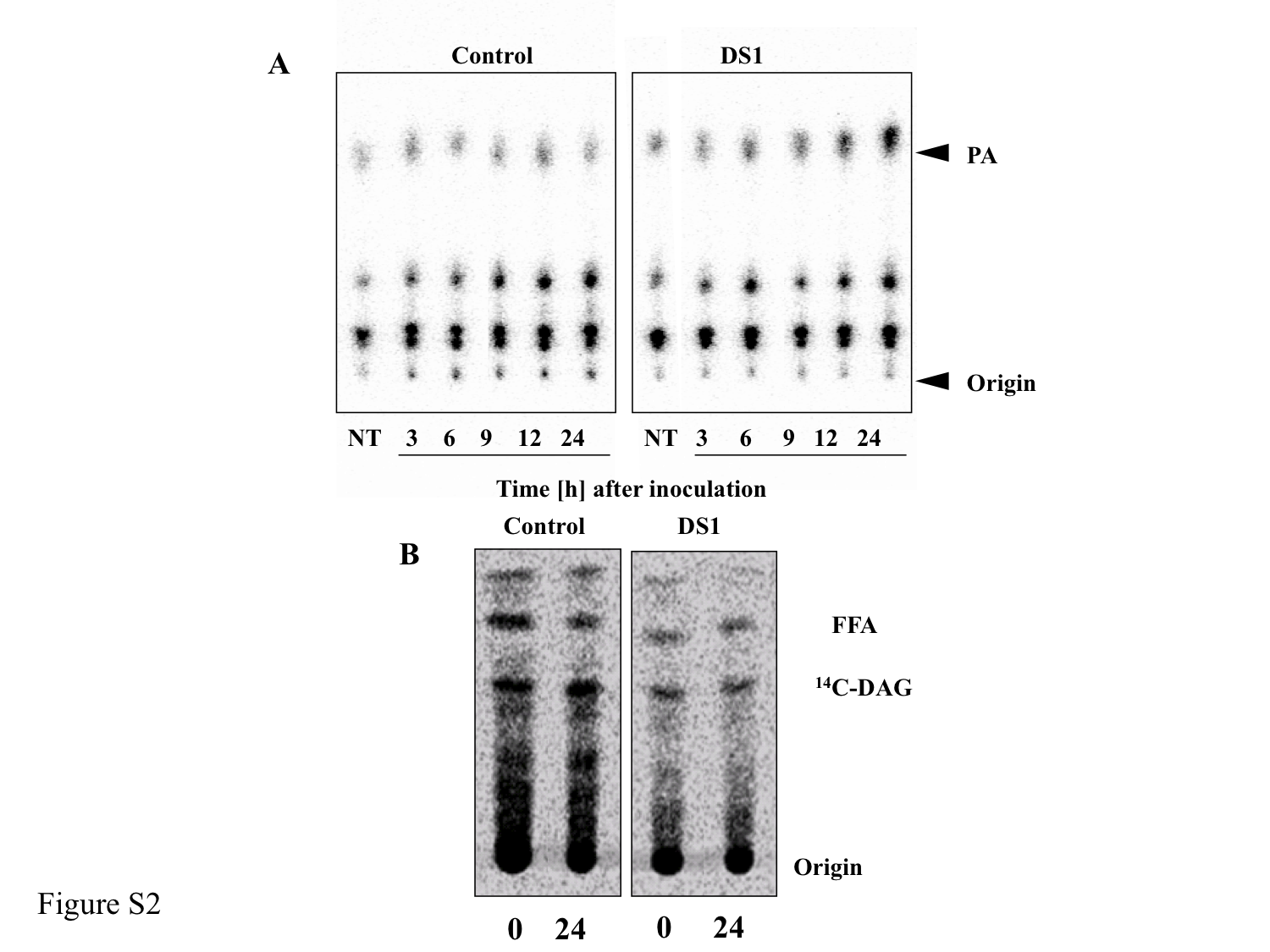

Supplement: Figure S2 — Separation and identification of phospholipids by TLC. Separation and identification of 32P-labeled PA (A) or C14-labeled diacylglycerol (B) by TLC. The migration of authentic phospholipid standards are indicated to the right of the TLC plate. (TIFF) [file pone.0075124.s002.tiff]

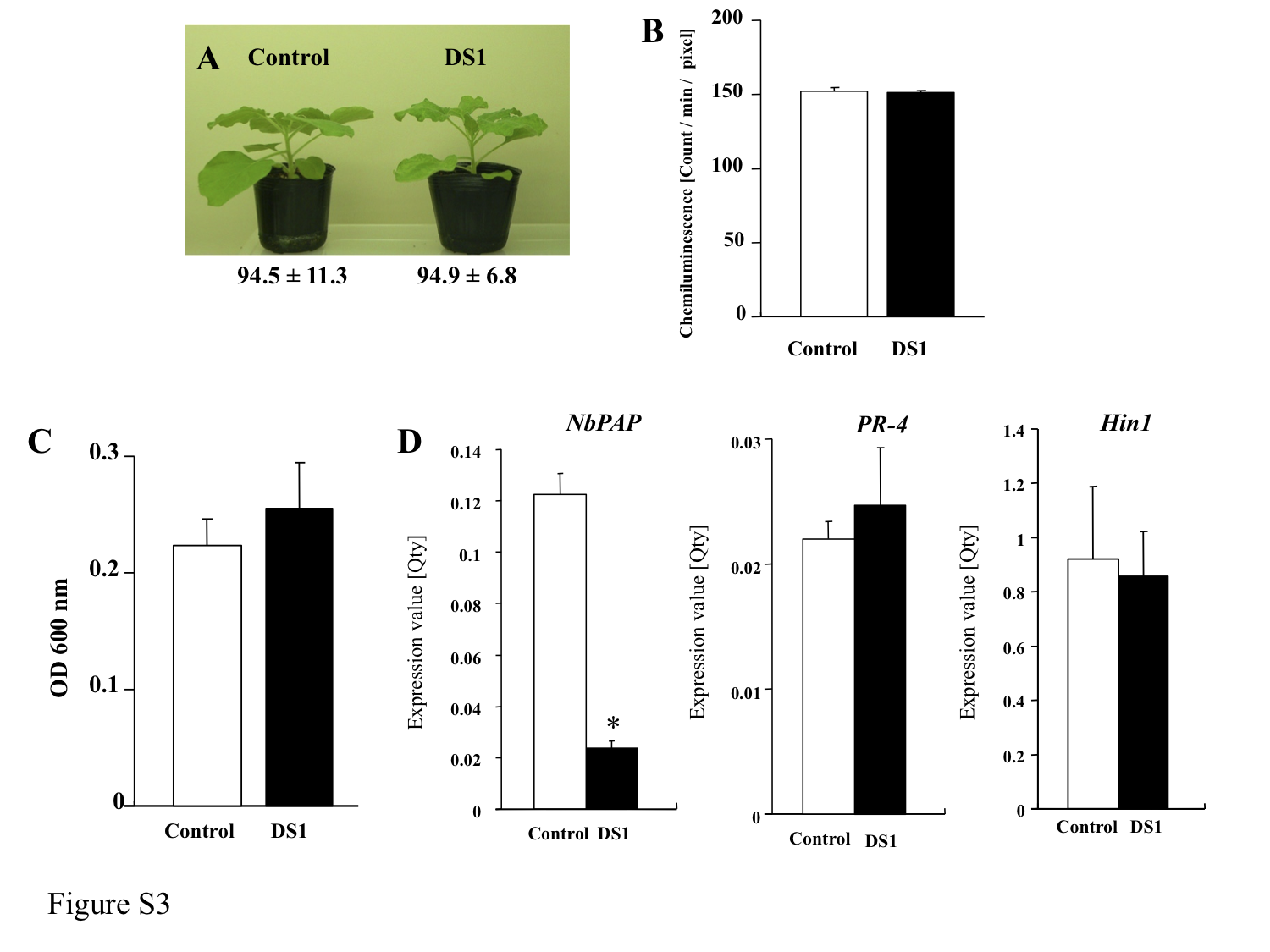

Supplement: Figure S3 — Phenotypic observation of DS1 plant. (A) Photograph was taken 3 weeks after inoculation with Agrobacterium tumefaciens. Values represent mean plant length (n = 7) with SD. (B) ROS production was determined by chemiluminescence as described in Materials and methods. (C) Cell death was determined by Evans blue staining. (D) Total RNA was isolated from control (Control) and DS1 plants (DS1). Transcript levels of DS1 were estimated by qRT-PCR. Values represent mean ± SD from triplicate experiments. (TIFF) [file pone.0075124.s003.tiff]

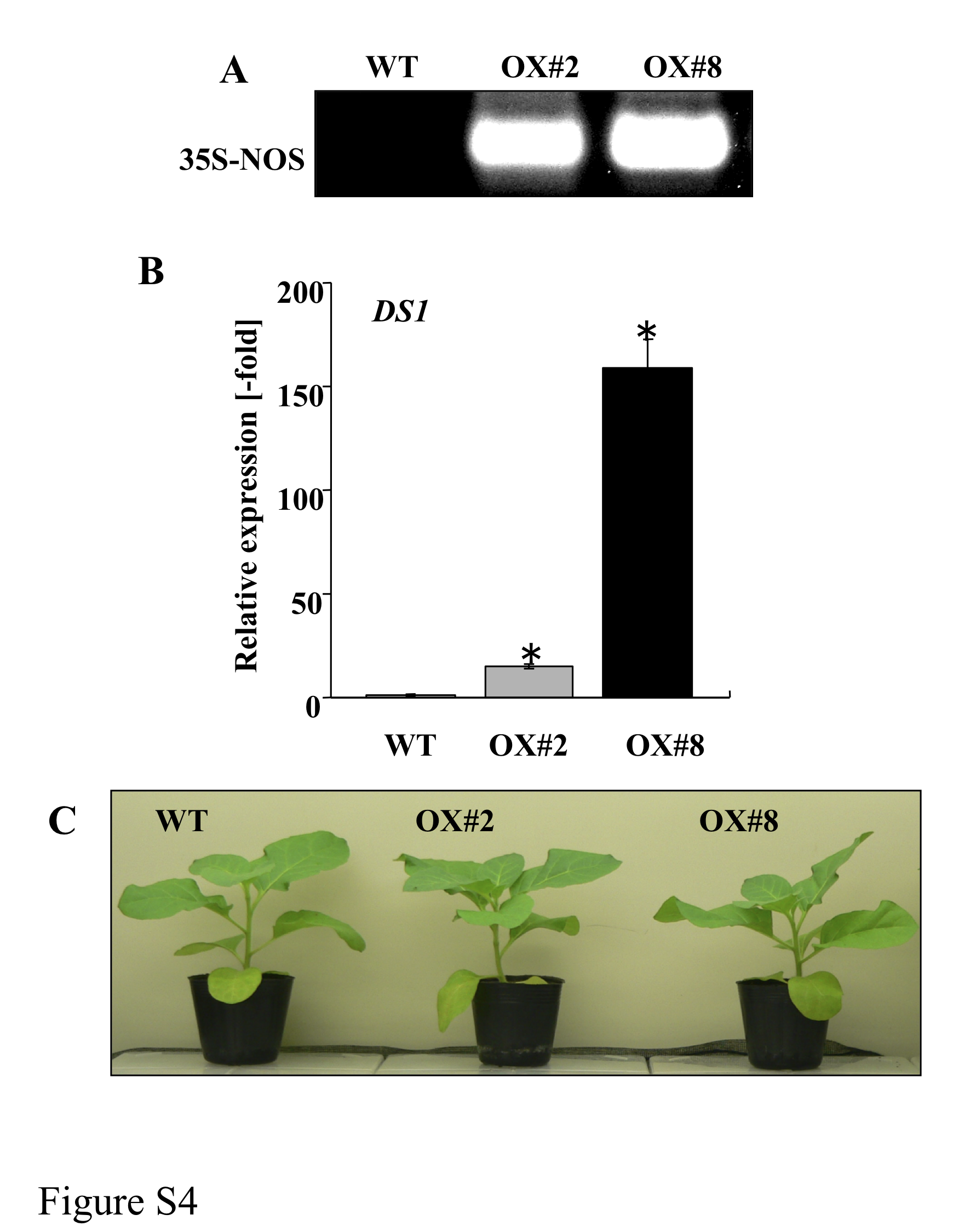

Supplement: Figure S4 — DS1-overexpressing transgenic tobacco. (A) Detection of DS1 gene in transgenic tobacco genome. Total genomic DNA was prepared from wild-type control (WT) or DS1-transformed plants (OX#2 and 8). Transformed DS1 was detected by RT-PCR using 35S promoter primer and NOS terminator primer. (B) Expression of DS1 in transgenic tobacco plants. Total RNA was isolated from fully expanded tobacco leaves of wild-type control (WT) or DS1-transformed plants (OX#2 and 8). Relative expression value of DS1 transcripts is shown to relative to that in wild-type control. Values are means of four replicate experiments with SD. Asterisks denote values significantly different from those of controls (*; P < 0.05, t-test). (C) Morphological observation of DS1-overexpressing plant. Photograph was taken 2 months after germination. (TIFF) [file pone.0075124.s004.tiff]

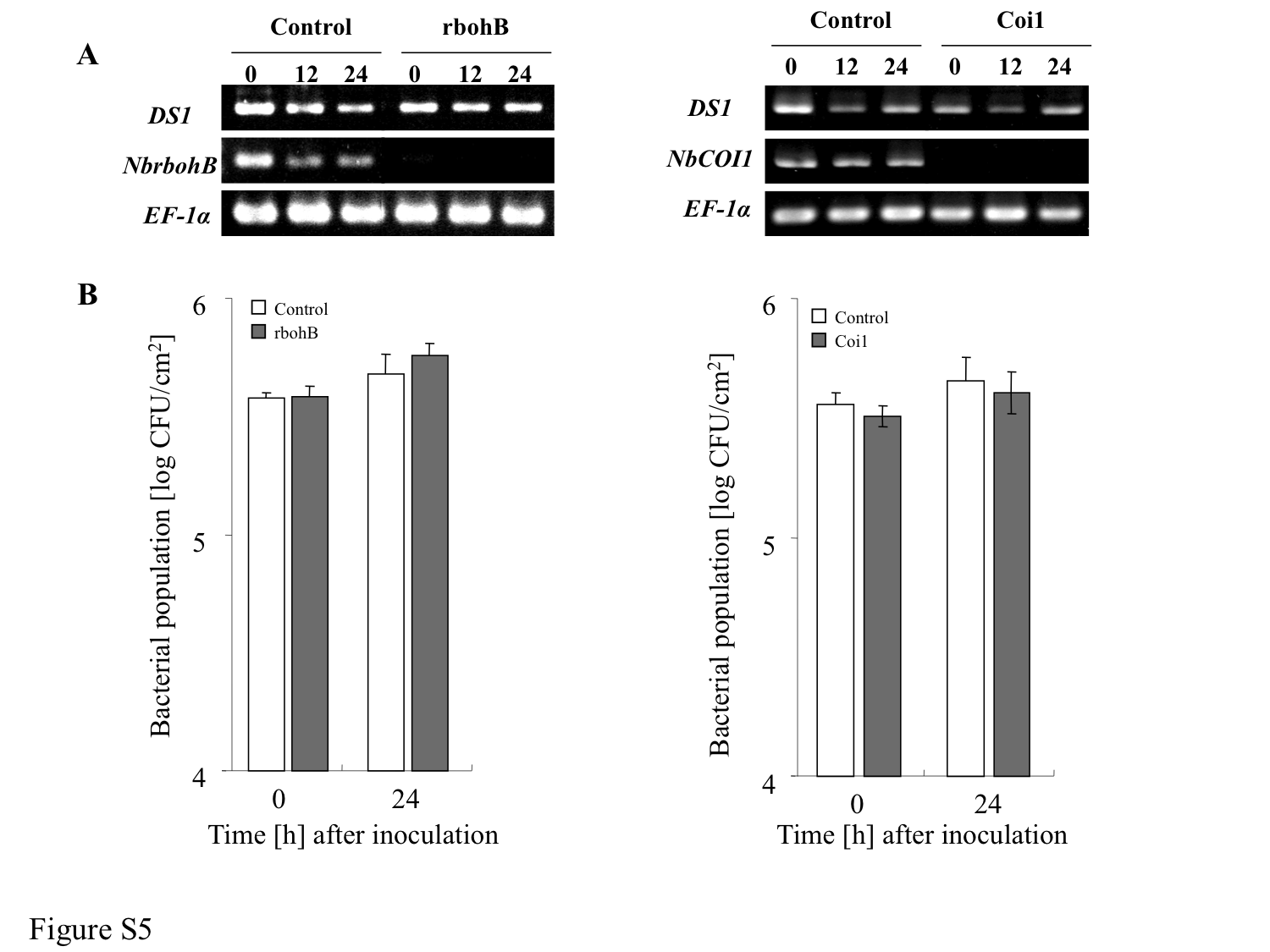

Supplement: Figure S5 — Phenotypic observation of NbrbohB and NbCoi1-silenced plants. (A) Total RNA was isolated from control, control, rboHB, and Coi1 plants 0, 12 and 24 h after inoculation with R. solanacearum. Semi quantitative RT-PCR was carried out with specific primers for NbCoi1 and NbrboHB. Equal loads of cDNA were monitored by amplifying constitutively expressed NbEF-1α. (B) Control and respective silenced plant leaves infiltrated with R. solanacearum (108 CFU/ml). Bacterial population was determined by plating at specified time points. Values are means of four replicate experiments with SD. Asterisks denote values significantly different from those of empty PVX controls (*; P < 0.05, t-test). (TIFF) [file pone.0075124.s005.tiff]

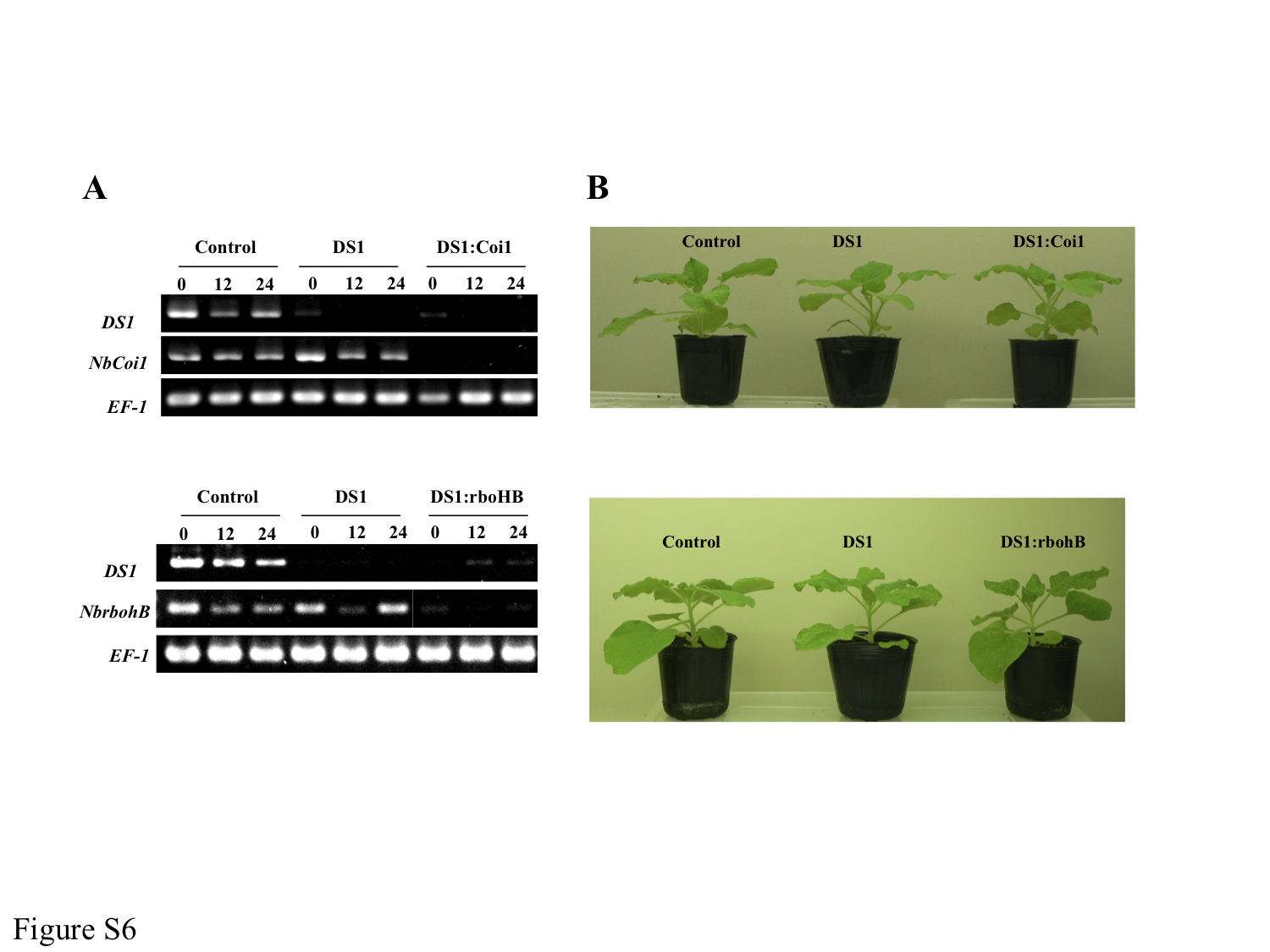

Supplement: Figure S6 — Phenotypic observation of double knock-down plants. (A) Total RNA was isolated from control, DS1, DS1:rboHB, and DS1:Coi1 plants 0–24 h after inoculation with R. solanacearum. Semi quantitative RT-PCR was carried out with specific primers for NbCoi1, NbrboHB, and DS1. Equal loads of cDNA were monitored by amplifying constitutively expressed NbEF-1α. (B) Control, DS1, DS1:rboHB, and DS1:Coi1 plants were photographed 3 weeks after inoculation with Agrobacterium tumefaciens. (TIFF) [file pone.0075124.s006.tiff]

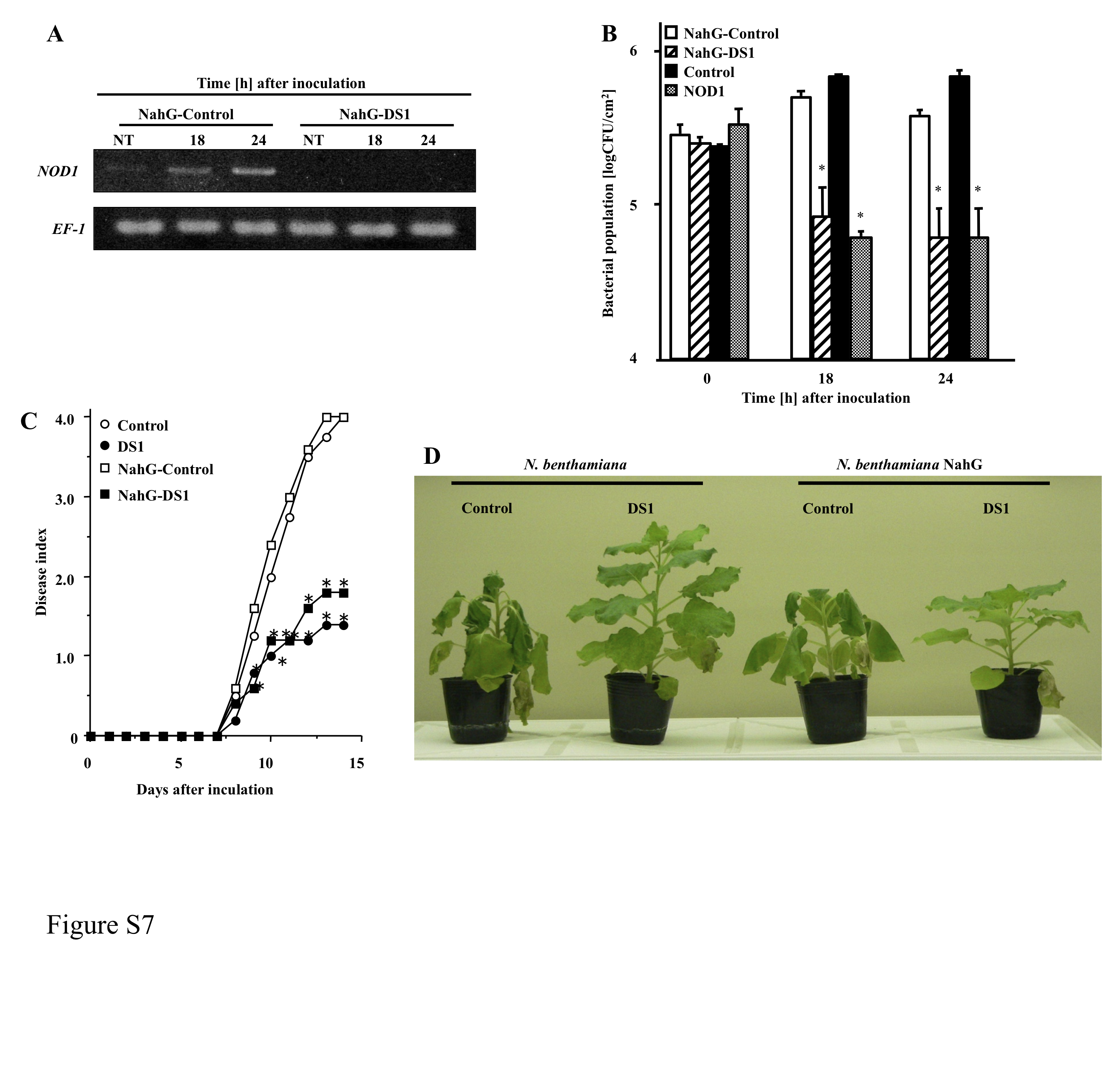

Supplement: Figure S7 — Role of salicylic acid in DS1 phenotype. (A) Total RNA was isolated from NahG and DS1-silenced NahG (DS1-NahG) plants 0–24 h after inoculation with R. solanacearum. Semi quantitative RT-PCR was carried out with specific primers for DS1. Equal loads of cDNA were monitored by amplifying constitutively expressed NbEF-1α. (B) Bacterial population was determined by plating at specified time points. Values are means of four replicate experiments with SD. Asterisks denote values significantly different from those of control (*; P < 0.05, t-test). (C) Disease development of bacterial wilt was rated daily on a 0–4 disease index in wild-type N. benthamiana control (Control), DS1 (DS1), NahG-control (NahG-Control), and NahG- DS1 (NahG-DS1) plants. Values are means of four replicate experiments with SD. Asterisks denote values significantly different from those of empty PVX control (*; P < 0.05, t-test). (D) Characteristic symptoms in control, DS1, NahG, and NahG-DS1 plants. Photograph was taken 12 days after inoculation with R. solanacearum. (TIFF) [file pone.0075124.s007.tiff]

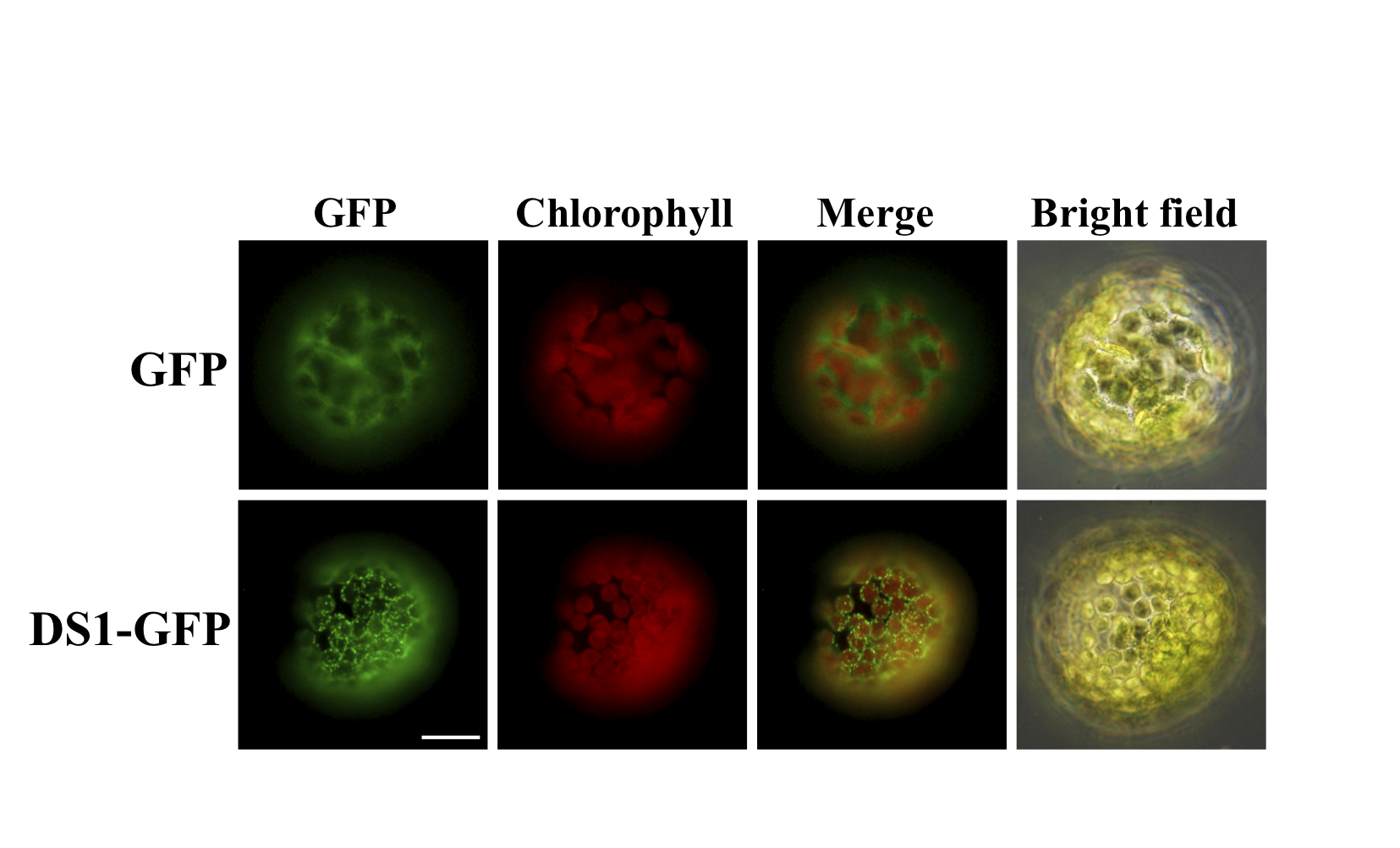

Supplement: Figure S8 — Subcellular localization of DS1. Localization of DS1-GFP in Nicotiana benthamiana protoplasts. Confocal images of protoplasts prepared from leaves inoculated with Agrobacterium tumefaciens carrying P35S-GFP (GFP) or p35S-DS1-GFP for 48 h. Observation of GFP fluorescence was carried out using the method described previously [62]. Scale bar represents 20 μm. (TIFF) [file pone.0075124.s008.tiff]

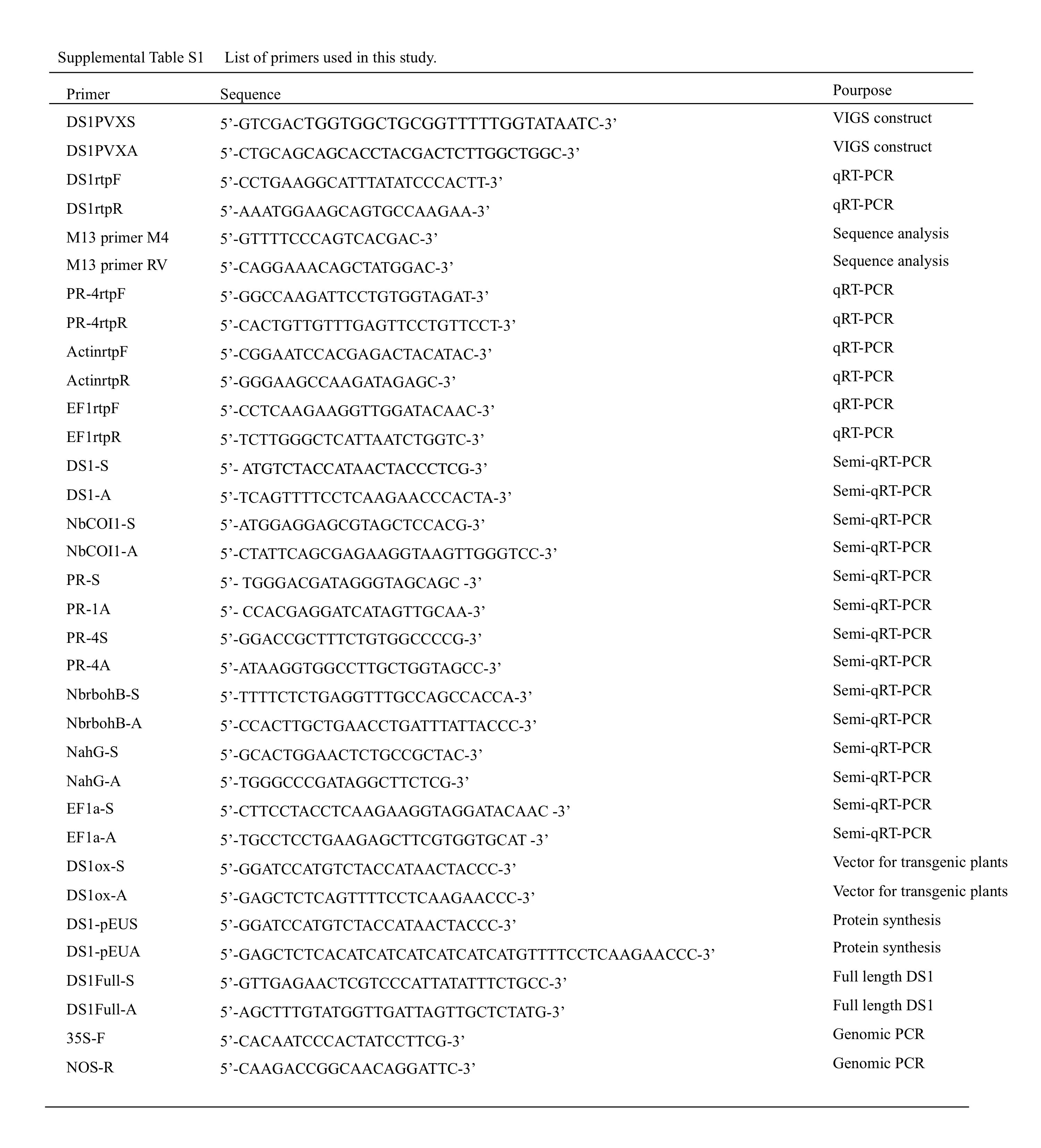

Supplement: Table S1 — List of primers used in this study. (TIFF) [file pone.0075124.s009.tiff]

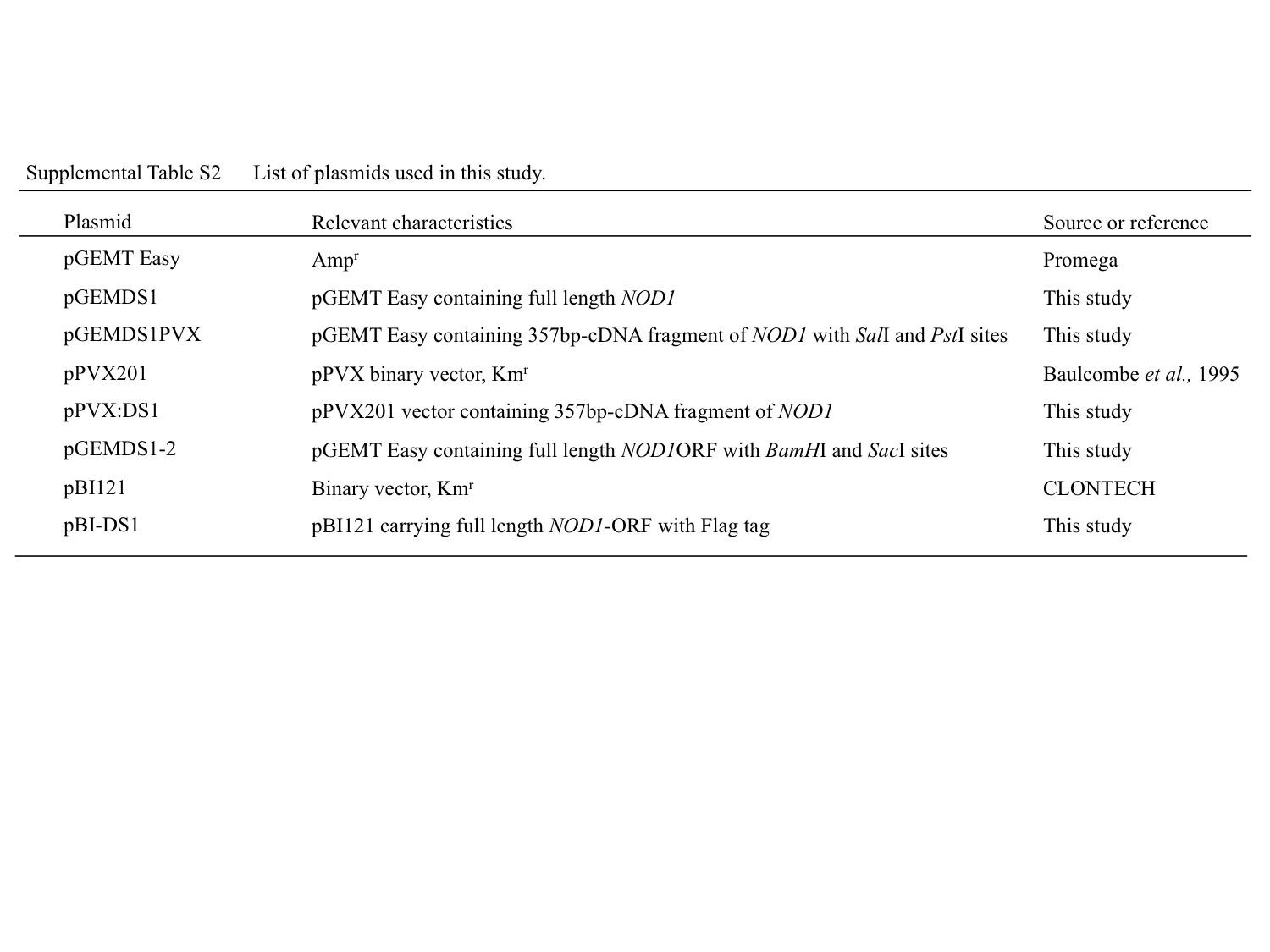

Supplement: Table S2 — List of plasmids used in this study. (TIFF) [file pone.0075124.s010.tiff]
